# New Library of Iodo-Quinoline Derivatives Obtained by an Alternative Synthetic Pathway and Their Antimicrobial Activity

**DOI:** 10.3390/molecules29040772

**Published:** 2024-02-07

**Authors:** Cristina Maria Al-Matarneh, Alina Nicolescu, Ioana Cristina Marinaş, Mădalina Diana Găboreanu, Sergiu Shova, Andrei Dascălu, Mihaela Silion, Mariana Pinteală

**Affiliations:** 1 Center of Advanced Research in Bionanoconjugates and Biopolymers, “Petru Poni” Institute of Macromolecular Chemistry of Romanian Academy, 41A Grigore Ghica Voda Alley, 700487 Iasi, Romania; idascalu@icmpp.ro (A.D.); pinteala@icmpp.ro (M.P.); 2 Research Institute of the University of Bucharest—ICUB, 91-95 Spl. Independentei, 050095 Bucharest, Romania; ioana.cristina.marinas@gmail.com (I.C.M.); gaboreanu.diana-madalina@s.bio.unibuc.ro (M.D.G.); 3 NMR Laboratory ”Petru Poni” Institute of Macromolecular Chemistry of Romanian Academy, 41A Grigore Ghica Voda Alley, 700487 Iasi, Romania; alina@icmpp.ro; 4 Department of Inorganic Polymers ”Petru Poni” Institute of Macromolecular Chemistry of Romanian Academy, 41A Grigore Ghica Voda Alley, 700487 Iasi, Romania; shova@icmpp.ro; 5 Physics of Polymers and Polymeric Materials Department, “Petru Poni” Institute of Macromolecular Chemistry, 41A Grigore Ghica Voda Alley, 700487 Iasi, Romania; silion.mihaela@icmpp.ro

**Keywords:** iodo-quinolines, Doebner synthesis, antimicrobial activity, *S. epidermidis*, *K. pneumonie*, *C. parapsilosis*

## Abstract

6-Iodo-substituted carboxy-quinolines were obtained using a one-pot, three-component method with trifluoroacetic acid as a catalyst under acidic conditions. Iodo-aniline, pyruvic acid and 22 phenyl-substituted aldehydes (we varied the type and number of radicals) or O-heterocycles, resulting in different electronic effects, were the starting components. This approach offers advantages such as rapid response times, cost-effective catalysts, high product yields and efficient purification procedures. A comprehensive investigation was conducted to examine the impact of aldehyde structure on the synthesis pathway. A library of compounds was obtained and characterized by FT-IR, MS, ^1^H NMR and ^13^C NMR spectroscopy and single-ray crystal diffractometry. Their antimicrobial activity against *S. epidermidis*, *K. pneumonie* and *C. parapsilosis* was tested in vitro. The effect of iodo-quinoline derivatives on microbial adhesion, the initial stage of microbial biofilm development, was also investigated. This study suggests that carboxy-quinoline derivatives bearing an iodine atom are interesting scaffolds for the development of novel antimicrobial agents.

## 1. Introduction

Since the majority of therapeutic medications are generated from heterocyclic structures, the synthesis of biologically active heterocyclic scaffolds has continuously drawn great interest in medicinal chemistry. This feature was revealed by Vitaku et al. in a remarkable review in 2014 [1], which showed that 85% of FDA-approved small-molecule drugs contain nitrogen, with 59% of them including at least one azaheterocycle. These are very high percentages, far higher than the effect figures previously revealed for fluorine (15%) and sulfur (26%). It is interesting to note that although the average amount of nitrogen atoms per medication for all small-molecule pharmaceuticals is 2.3 N/drug, it is 35% higher for drugs that include a nitrogen heterocycle (3.1 N/drug). In this context, the quinoline nucleus is present in a wide variety of synthetic and natural azaheterocyclic products with intriguing biological characteristics [2]. Although quinoline itself possesses few applications, many of its derivatives are useful in a variety of domains, including pharmaceuticals and advanced functional materials, one example being fluorescent probes containing a quinoline-based core [3,4]. Quinoline-containing compounds have a well-established pharmacological value, starting with the famous chloroquine, used for decades to control and eradicate malaria. Other currently available drugs containing these privileged moieties are antimalarials (quinine, quinidine, mefloquine, amodiaquine, primaquine and so on), antiviral (saquinavir), antibacterial (fluoroquinolones such as ciprofloxacin, sparfloxacin, gatifloxacin and so on), antifungal–antiprotozoal (clioquinol) or anthelmintic (oxamn and vesnarinone) [5]. Since the structural change of a favored moiety with therapeutic ability significantly alters its therapeutic value, the development of novel synthetic medicines based on the quinoline scaffold continues to be an active research topic.

**Figure 1:**
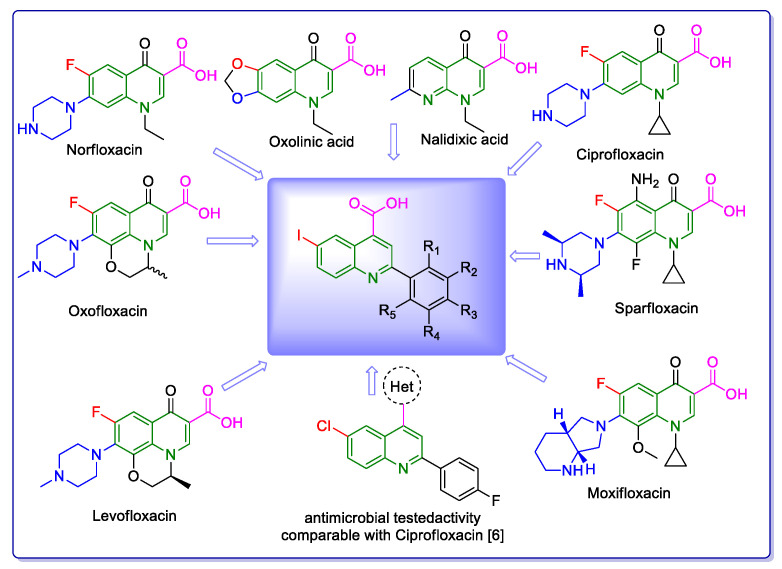
Rational design used in this study to obtain iodine carboxy-quinolines.

The need to discover novel antimicrobial agents with better properties and fewer side effects is a continuous preoccupation in the medical community. When it comes to different Gram-positive and Gram-negative bacteria species, the majority of quinoline compounds show good antimicrobial action, determined by the substitution of the heterocyclic pyridine ring rather than the aromatic moiety [6]; some examples in this sense are presented in Figure 1. Unfortunately, the widespread overuse of quinoline-based drugs led to an increased occurrence of antimicrobial resistance. As a result, several well-established synthetic techniques for creating novel, appropriately substituted quinoline scaffolds were developed over time: Combes, Skraup, Döbner–Von Miller, Conrad–Limpach, Pfitzinger, Friedländer and Povarov syntheses or the homogeneous metal-catalyzed hetero-annulation of acyclic precursors [7].

One of the most significant groups of quinoline derivatives is quinoline-4-carboxylic acids, which are used as active ingredients in commercial antioxidants and have a range of therapeutic uses [8]. In order to obtain physiologically active carboxy-quinolines, the widely used reactions are Doebner synthesis (uses activated substituted amines, aldehydes and pyruvic acid) and Pfitzinger condensation (the reaction of isatins with α-methylidene carbonyl compounds) [9]. These classic synthetic approaches suffer from limited sources of precursors, harsh reaction conditions, low yields or selectivity and difficult operations. Thus, modified approaches of these classical syntheses are still of great value, especially if they can be more efficient and easy to operate at a lower cost. 

In light of this, keeping with our quest for physiologically active hybrid heterocyclic molecules [10,11], as part of our concern for microbial active compounds [12,13,14,15], we present the synthesis of a library of novel iodine carboxy-quinoline derivatives as well as the resulting by-products. For this series of compounds, we looked at the influence of the electronic effects of the starting components and structure, and we evaluated the in vitro antibacterial activity.

## 2. Results and Discussion

### 2.1. Chemistry

**Figure 2:**
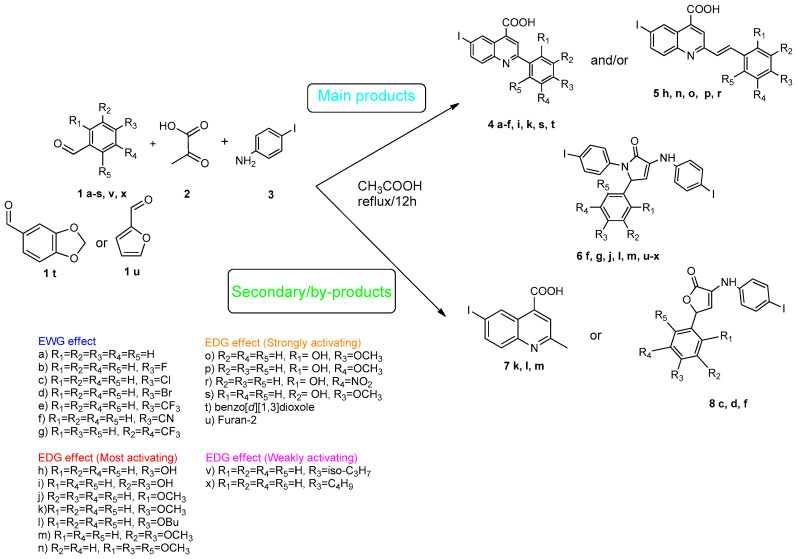
Quinoline-based compound synthesis.

Povidone-iodine is a topical antiseptic medication used for wound therapy and infection prevention. It is a chemical compound of polyvinylpyrrolidone and triiodide [16]. Carboxy-quinolines substituted in position 5 with withdrawing radicals are mainly obtained through the Pfitzinger condensation of an appropriate isatin and α-methylidene carbonyl compounds in the presence of potassium hydroxide in aqueous ethanol under reflux followed by neutralization. In our previous study [16], we found that using the Doebner reaction conditions (ethanol, TFA as a catalyst and reflux for 12 h), we obtained pyrrole-2-one derivatives along with a few by-products. In this investigation, we changed the solvent conditions with acetic acid while keeping the other conditions constant. As a result, we successfully obtained quinoline derivatives. In addition to the desired quinoline derivatives, we separated from the reaction mixtures either pyrrole-2-ones, furan derivatives or methyl carboxy-quinoline as the major product, as presented in Figure 2. Based on these findings, we concluded that the main factors influencing the reaction pathways were the electronic effects and position of substituents in the starting aldehydes [17]. 

**Figure 3:**
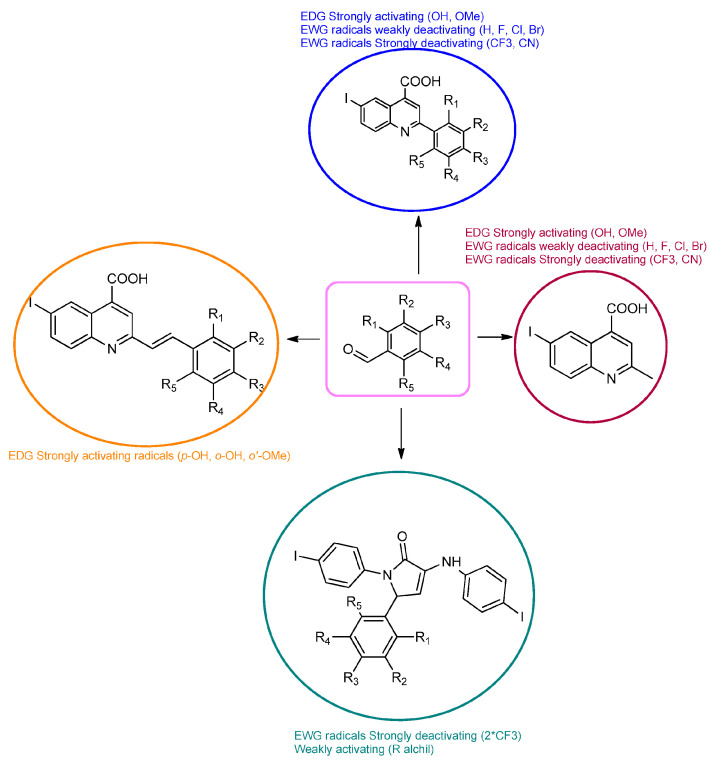
Reaction products depending on aldehyde substituents.

Therefore, in the presence of an electron-withdrawing group at the *para* position of the aldehyde reactant (**1c**, **d**, **f**), the main product generated is carboxy-quinolines (**4a**–**f**), along with **6f** or/and **8c**, **d**, **f** as by-products. These additional structures are formed when the substituents are strongly deactivating, such as halogen and cyan groups. By introducing an additional electron-withdrawing group and repositioning them to the *meta*, *meta’* locations, the pyrrole-2-one derivative **6g** was exclusively obtained. Moving to the electron-donating group (weakly activating in the case of aldehydes **1v** and **1x**), we separated the same pyrrole-2-one derivatives (**6v** and **6x**). In the case of the strongly activating electron-donating group, we obtained different results depending on the position and/or combination of substituents. Therefore, carboxy-quinolines were produced when the methoxy group was the lone substituent in the *para* position (**4k**) or when the *para* position was occupied by the hydroxy or methoxy group and the *meta* position was occupied by the hydroxy group (**4s**). When a sterically hindered group was used such as butoxy that occupied the *para* position, a pyrrole-2-one derivative and a quinoline methyl substituted as a secondary product were formed. Once the **1m** reactant, containing two methoxy groups, was applied in the reaction, we did not achieve the intended formation of quinoline, but instead, the pyrrole derivative **6m** was produced. When the number of methoxy substituents in the aldehyde reactant is increased to 3 (**1n**), it leads to carboxy-quinoline derivatives (**5n**) containing an ethylenic bridge between the quinolinic and aldehyde cores. Similar compounds (**5o**, **5p**, **5r**) were formed when the initial aldehyde had two substituents. During a particular experiment, the replacement of the *para* group with a hydroxyl group, which is a powerful electron donor, led to the formation of a derivative of the same type (**5h**). For oxygen heterocycle aldehydes **1t** and **1u**, we noticed that the inclusion of a phenyl core led to the stability of the structure, resulting in the formation of **4t** quinoline. Specifically, derivative **1u** mostly produced the chemical pyrrole-2-one **6u**. A schematic representation of the electronic effect on the final compound’s formation is presented in Figure 3, and a detailed table with the obtained products and by-products is presented in Table 1. 

The structures of the newly synthesized compounds have been established by employing spectral (NMR, IR) mass spectrometry and X-ray diffraction studies (details are presented in the Section 3 and Figures S1–S10 from the Supplementary Information).

Details on proton and proton–carbon spin systems were acquired using homo- and heteronuclear bidimensional correlation experiments such as H, H-COSY, H,C-HSQC and H,C-HMBC. In the ^1^H-NMR spectra, the formation of the carboxy-quinoline core is indicated by the presence of four signals within the range of 7.80–9.20 ppm, with splitting patterns dependent on the proton–proton scalar couplings: doublet at 7.90 ppm, doublet of doublets at 8.10 ppm, singlet at 8.50 ppm and another doublet at 9.14 ppm. The proton of the carboxylic group presents a very broad resonance signal centered at around 14 ppm. Quinoline’s aromatic substituent has proton resonance signals in the same region, their number and multiplicity being determined by the structure of the starting benzaldehyde. Thus, the presence of *para*-substituted benzaldehyde moieties in the reaction products **4b**–**f** and **4k** are recognized after the two doublets with a roof-effect pattern, whereas the moieties of disubstituted benzaldehydes have resonance signals as two doublets with 2 and 8 Hz coupling constants and a doublet of doublets (**4i** and **4s**). In the case of fluorine-substituted benzaldehyde moieties (**4b**), the additional proton–fluorine couplings alter the splitting pattern, generating a triplet and a doublet of doublets. The ethylenic bridge identified in quinoline derivative **5** was deduced from the presence in the proton spectra of two doublets with 16 Hz coupling constants, resonating in the interval 7.30–8.15 ppm. Both the coupling constants and chemical shift values are in agreement with this proposed vinylic group, indicating a *trans* proton configuration. The corresponding carbon atoms were identified in the 120–130 ppm interval in ^13^C-NMR spectra, where double-bond carbon atoms usually resonate. Three-bond proton–carbon correlations identified in the HMBC spectrum support the covalent link of the ethylenic group with carboxy-quinoline and benzaldehyde cores. Both vinylic protons present correlation signals in the long-range HMBC spectrum with either quinoline’s CH-3 or benzaldehyde’s C-14 carbon atoms, as exemplified in Supporting Information Figure S4 for derivative **5o**. 

The formation of side products with pyrrole-2-one (**6**) or furan-2-one (**8**) cores was readily seen in the proton NMR spectra. In the case of these derivatives, the carboxy-quinoline characteristic ^1^H-NMR pattern was replaced by two doublets with 3 Hz coupling constants, resonating at around 6.0–7.0 ppm, previously assigned by us as fast NMR indicators for the formation of pyrrole-2-one or furan-2-one cores [17]. The rest of the proton signals present in the spectra of side products were also in good agreement with our previous NMR description of a series of iodo-dihydro-pyrrole-2-one derivatives [17]. 

The presence in the proton spectra of a carboxy-quinoline NMR pattern, with a new singlet at 2.70 ppm in the absence of benzaldehyde characteristic signals, clearly indicated a carboxy-quinoline substituted only with methyl, as exemplified in Figure S5 from the Supporting Information for compound **7**.

The unambiguous proton and carbon signal assignments for all new compounds, obtained from bidimensional correlation experiments, are presented in the Section 3.

The IR spectra of the obtained compounds show absorption bands specific for both aromatic and aliphatic C-H bonds, indicating the presence of the N-heterocycles (Section 3 and Figures S1–S10 from Supporting Information). The bands above 3300 cm^−1^ can be attributed to NH bonds and OH groups depending on the structure of the molecule being studied, while the bands related to C-N stretching bonds are in the range of 1335–1240 cm^−1^ and the bands specific to the absorption of ketone carbonyl groups are in the range of 1700–1650 cm^−1^. All the other observed bands are in good agreement with the proposed structure [17].

The interaction between iodo-aniline, aldehyde derivatives and pyruvic acid with the formation of compounds **4**, **5**, **6**, **7** and **8** was also confirmed by MALDI-MS analysis (Section 3 and Figures S1–S10 from Supporting Information). Only positive ions were studied because the molecular ion only forms in the positive mode. By using the aforementioned method, we were able to achieve strong correlations between estimated mass and measured mass for each molecule, confirming all posited structures. 

**Figure 4:**
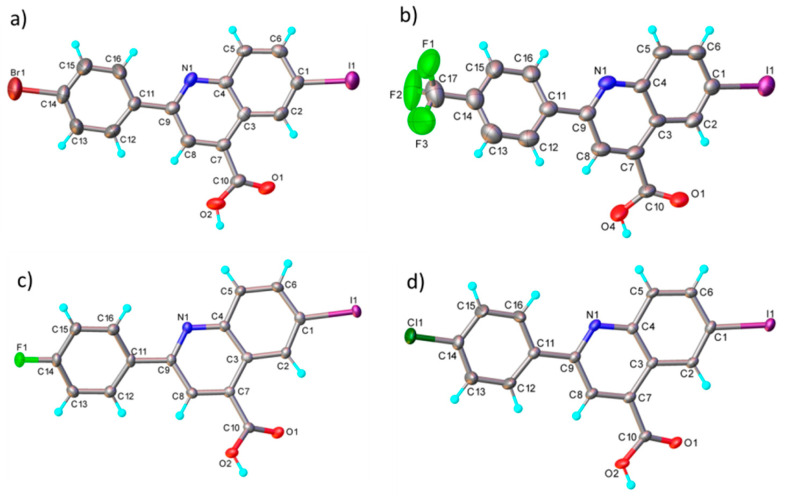
X-ray molecular structure of **4d** (**a**), **4e** (**b**), **4b** (**c**) and **4c** (**d**) with atom labeling and thermal ellipsoids at 50% level.

The structure and chemical composition of compounds **4b**, **c**, **d** and **e** were also confirmed by the single-crystal X-ray diffraction method (full information can be found in Tables S1–S5 from the Supporting Information). Accordingly, it was demonstrated that the compounds have a molecular crystal structure comprising neutral units, as illustrated in Figure 4 (**4d** (a), **4e** (b), **4b** (c) and **4c** (d)). It is to be noted that compounds **4c** and **4d**, which crystallize in the P21/n space group with very close unit cell parameters, are essentially planar, while for **4b** and **4e,** the phenyl and quinoline nuclei are rotated by 20.9(2)° and 22.5(1)°, respectively. This is caused not by the presence of steric effects but due to the peculiarities of the crystal structure packing. Further analysis has shown that the crystal packing of **4c** and **4d** is also similar, being driven by the presence of C-H···O, π-π stacking and halogen intermolecular contacts, which is illustrated in Figure S6.

In the crystals of **4b** and **4c,** the asymmetric units are assembled to form a dense three-dimensional supramolecular network. As an example, a view of the crystal packing along the *b*-axis for **4d** is shown in Figure S7. The results of the X-ray diffraction study for compounds **4b** and **4e** are shown in Figure S8. It is to be noted that, as a whole, the system of intermolecular interactions in crystals **4e** and **4b** resembles well that observed for the above-mentioned **4d** and **4c**. As a result, the crystal structure of **4e** and **4b** is also characterized as a 3D supramolecular architecture, as depicted in Figure S9. 

**Figure 5:**
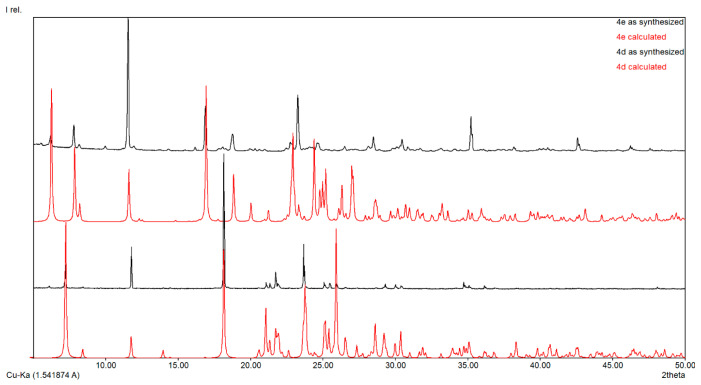
Powder XRD of compounds **4d** and **4e**.

Important data about purity are revealed by powder X-ray diffraction pattern (PXRD) analysis of the produced substances. The PXRD of the synthesized compounds **4d** and **4e** are shown in Figure 5 and for compounds **4b** and **4c** in Figure S10. The diffraction peaks of the synthesized compounds are in good agreement with the simulated data.

### 2.2. Antimicrobial Activity

The most commonly used quinolones (ciprofloxacin, norfloxacin, oxofloxacin and levofloxacin) contain in their chemical structures, in addition to a quinolone center, a carboxyl group and a fluorine atom in position 5. In this study, we kept the carboxyl group in our structures and replaced fluorine with iodine (in the same position) in order to obtain better antimicrobial properties, as iodine insertion in organic molecules increases the antimicrobial activity against *S. aureus* [18,19,20].

Minimum inhibitory concentration (MIC) values were determined by the binary microdilution method. The results of the iodo-quinoline derivatives were compared with the basic structure and the solvent used (Table 2). Based on the information provided in Table 1, all compounds exhibited antibacterial effects against *S. epidermidis*, had no effect on *K. pneumoniae* and showed varying degrees of antifungal activity against *C. parapsilosis*. It is evident that the compounds exhibit varying levels of antibacterial and antifungal action based on the radicals linked to the basic structure.

**Figure 6:**
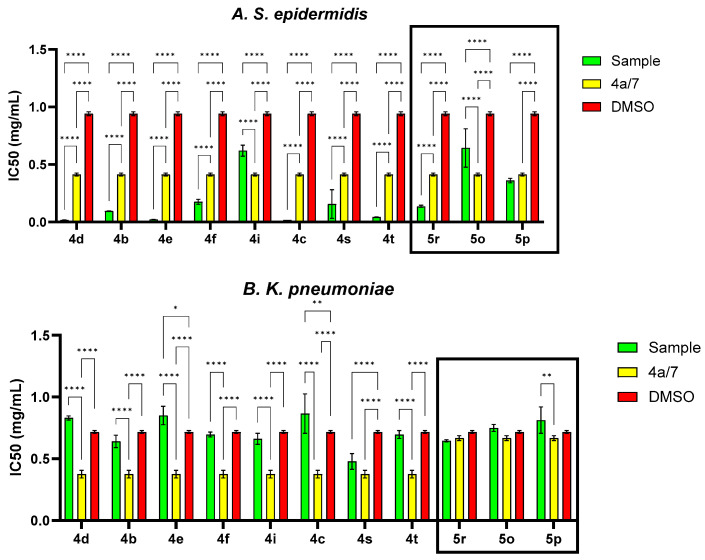

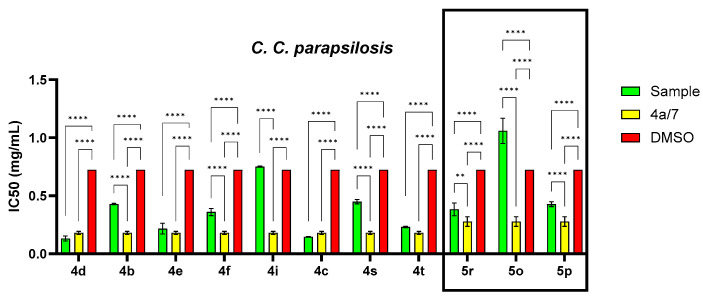
IC50 values (mg/mL) for each compound tested related to the general structure and solvent control for *S. epidermidis*, *K. pneumoniae* and *C. parapsilosis* strains (* *p* < 0.05, ** *p* < 0.01, **** *p* < 0.0001).

In order to evaluate the antimicrobial activity improvement, the statistical analysis was carried out following the general structure, and the IC50 (percent inhibition is 50%) was determined for each variant (Figure 6). Therefore, the pattern of antimicrobial activity based on the IC50 against Gram-positive bacteria (*S. epidermidis*, Figure 6) may be observed as follows: in the quinoline series, the order of compounds from higher to lowest reactivity is **4d** > **4c** > **4e** > **4t** > **4b** > **4s** > **4f** > **7** > **4a** > **4i** > **DMSO** and in styryl quinoline series is **5r** > **5p** > **5o** > **DMSO**. The presence of the -C_6_H_4_Br group in the basic structure exhibited the highest level of activity towards the basic one, whereas the presence of the -C_6_H_4_Cl group in the same initial structure showed a somewhat lower level of activity. The initial structure (**7**) and the basic one (**4a**) exhibit notably higher efficiency against *S. epidermidis* than DMSO. Derivatives from the basic structure (**4a**) show significantly higher activity compared to the **5o, 5p** and **5r** variants.

Regarding Gram-negative bacteria such as *K. pneumoniae*, it can be concluded that the MIC values are determined by DMSO (Table 2). In some cases, DMSO even enhanced cell proliferation (**4e** and **4a**), resulting in higher values compared to those attributed to the solvent. Analyzing the IC50 values (Figure 6), it is evident that the basic structure exhibited a substantially lower IC50 value in comparison to the majority of the derivatives. This observation indicates that the antibacterial effectiveness of quinoline derivatives was ineffective against Gram-negative bacteria, perhaps due to the distinctive outer membrane of these cells. Resistance may arise due to mutations in porins or other constituents, alterations in the hydrophobic nature of the outer membrane or other modifications induced in Gram-negative bacteria. Gram-negative bacteria exhibit greater resistance compared to Gram-positive bacteria due to the absence of a critical membrane in Gram-positive bacteria [21]. The pattern of antibiotic efficacy against *K. pneumoniae*, as measured by IC50, was as follows: for the quinoline series, it is **4a** > **4s** > **4b** > **4i** > **7** > **4f** > **4t** > **DMSO** > **4d** > **4e** > **4c**, and for the styryl quinoline series, it is **5r** > **DMSO** > **5o** > **5p**. By comparing the IC50, it can be seen that the IC50 value for the basic structure was significantly lower compared to most of the derivatives. This aspect suggests that the antibacterial activity of quinoline derivatives was not active on Gram-negative bacteria, probably due to the specific outer membrane of these cells. Resistance can result from mutations in porins or other components, changes in the hydrophobicity of the outer membrane or other modifications triggered in Gram-negative bacteria. Gram-negative bacteria are more resistant than Gram-positive bacteria because Gram-positive bacteria lack this crucial membrane [22]. The trend of antimicrobial activity against *K. pneumoniae*, according to IC50, was as follows: **4a** > **4s** > **4b** > **4i** > **7** > **4f** > **4t** > **DMSO** > **4d** > **4e** > **4c** for quinoline series and **5r**> **DMSO** > **5o** > **5p** for styryl quinoline series.

The antifungal activity against the fungus *C. parapsilosis* (Figure 6), as measured by IC50, showed no significant variations between the derivatives and the original compound. The antifungal activity exhibited a significant difference compared to the solvent control (*p* < 0.0001, except for **4i** (*p* > 0.05)). The trend observed for the quinoline series was **4d** > **4c** > **4a** > **4e** > **4t** > **7** > **4f** > **4s** > DMSO > **4i**, and for the styryl quinoline series, it was **5s** > **5p** > DMSO > **5r.**

The compounds that presented an MBC value lower than the solvent were considered active. The MBC value is usually greater than or equal to the MIC value. From Table 2, it can be seen that in the case of *S. epidermidis*, the variants **4d**, **4a**, **4b**, **4e**, **4f**, **4c**, **4t** and **7** have a better microbicidal effect than the solvent used, the most active being **4s**, **4c**, **4e** and **4d**. In acute wounds, *S. epidermidis* is the most common strain [23]. Furthermore, *S. epidermidis* is not just a skin contaminant; its peptides contribute to the growth of pathogenic bacteria and might be a source of genes that confer antibiotic resistance [21]. Gram-negative bacteria were more frequently identified from chronic wounds than Gram-positive bacteria [24]. It is advised to utilize these compounds as active principles for formulations intended to treat acute wounds because they do not exhibit bactericidal action on Gram-negative bacteria, like the *K. pneumoniae* strain. According to Dowd et al. [25], the two most common species of yeast found in wounds were *C. albicans* and *C. parapsilosis*. We evaluated only the *C. parapsilosis* strain in this investigation to compare the antifungal activity of all iodo-quinoline derivatives. Table 2 reveals that only the variants **4c**, **4s** and **4t** have a fungicidal impact against *C. parapsilosis*; the rest of the compounds’ action is given by DMSO unless they have only fungistatic activity. 

Self-preservation has evolved in all microbes through parasitic connections in the form of biofilms. Biofilms are made up of a complex protective glycocalyx that bacterial colonies generate to defend themselves from host defense and antimicrobial treatment [26]. Biofilms are found in chronic and acute wounds, where they provide a risk of delayed infection. This process can also induce persistent and recurring skin infections [27].

**Figure 7:**
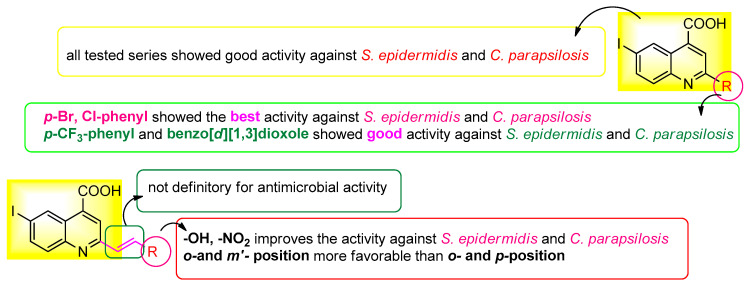
Antimicrobial efficiency diagram of the tested scaffolds against the three tested strains.

The development of novel compounds capable of inhibiting biofilm formation might be one of the solutions to both the rise in antibiotic resistance and the development of hard-to-heal wounds [28]. In this regard, the effect of iodo-quinoline derivatives on microbial adhesion, the initial stage of microbial biofilm development, was investigated. The values of the minimum inhibitory concentrations for microbial adhesion (MICMA) obtained are shown in Table 2. Compounds with MBEC values less than MIC are deemed active on microbial adhesion. The majority of the substances prevent the adherence of *S. epidermidis* cells. The compounds with greater activity related to both the basic structure (**4a**) and DMSO include **4d**, **4t**, **4e** and **4c**, while the active derivative from the **7** structure was only the **5r** variant. In the case of the **4a** base structure, a decrease in microbial adherence was also seen for the *K. pneumoniae* strain. The compounds with greater activity against *C. parapsilosis* cell adherence related to both the basic structures (**4a** and **7**) and DMSO include **4d**, **4t** and **4c**. A structure-antimicrobial efficiency diagram is presented in Figure 7.

## 3. Materials and Methods

Analytical thin-layer chromatography was performed with commercial silica gel plates 60 F_254_ (Merck Darmstadt, Germany) and visualized with UV light (*λ*_max_ = 254 or 365 nm). 

The ^1^H and ^13^C NMR spectra recorded for this study were obtained for solutions in DMSO-d6 at 298 K on Bruker Avance NEO 400 and 600 MHz spectrometers, equipped with a 5 mm four nuclei direct detection z-gradient probe (H,C,F,Si-QNP) and 5 mm inverse detection multinuclear z-gradient probe, respectively, with solvent peaks as reference. All chemical shifts were reported in ppm (δ), and coupling constants (*J*) were reported in Hertz (Hz). All the assignments were confirmed by information from proton–proton and proton–carbon two-dimensional NMR correlation experiments (COSY, HSQC and HMBC).

IR spectra were recorded on a Shimadzu IRTracer-100 instrument (Shimadzu U.S.A. Manufacturing, Inc., Canby, OR, USA). The melting point of the compounds was measured on MEL-TEMP capillary melting point apparatus from ambient temperature up to 400 °C. All commercially available products were used without further purification unless otherwise specified.

Mass spectra were acquired on a Bruker RapifleX MALDI-TOF/TOF (Bruker Daltonics, Bremen, Germany) equipped with a Smartbeam 3D laser. The FlexControl Version 4.0 and FlexAnalysis Version 4.0 software (Bruker, Bremen, Germany) were used to control the instrument and process the MS spectra.

The samples were initially dissolved in DMSO and subsequently diluted 10-fold in methanol. For the MALDI matrix solutions, 20 mg of α-cyano-4-hydroxycinnamic acid (HCCA) was dissolved in 1 mL of methanol. Subsequently, the MALDI matrix solution and the sample solution were mixed in a 2:1 ratio, and finally, 1 µL from each resulting solution was deposited onto the MALDI target and dried at room temperature prior to MALDI-MS analysis. Mass calibration of MALDI-TOF/TOF-MS was carried out using the peptide mixture standard solution (Bruker Daltonics, Bremen, Germany).

FlexControl 4.0 was used to optimize and acquire data using the following parameters: positive ion polarity in reflector mode, mass scan range *m*/*z* 100–1600 Da), digitizer 1.25 GHz, detector voltage 2117 V, 1000 shots per pixel and 5 kHz laser frequency. The laser power was set at 60% to 80% of the maximum and 1000 laser shots were accumulated for each spectrum.

### 3.1. General Procedure for Synthesis of Compounds ***4***, ***5***, ***6***, ***7*** and ***8***

Aldehydes **1a**–**v** (1 mmol) were solubilized in a minimum amount of acetic acid. A mixture containing pyruvic acid (1,5 mmoli) and TFA (20 µL) as a catalyst was then added to acetic acid and stirred for 10 min. Finally, iodo-aniline (1 mmol) was dissolved in a minimum amount of acetic acid and added and the resulting mixture was left to reflux for 12 h. The required products were obtained by filtering out the resulting suspension and washing the solid with ethanol. Dichloromethane and ethanol were used to facilitate recrystallization.


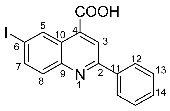


**6-iodo-2-phenylquinoline-4-carboxylic acid 4a**: crystallized from acetic acid; brown powder; 79% yield; mp 282–285 °C; IR ATR ν(cm^−1^): 3650, 3321, 3271, 3074, 2982, 2971, 2886, 1690, 1653, 1597, 1541, 1512, 1482, 1443, 1375, 1328, 1259, 1233, 1154, 1089, 1021, 952, 864, 764, 677, 587.

^1^H NMR (600.1 MHz, DMSO-d_6_, δ (ppm)): 7.56 (t, ^3^J = 7 Hz, 1H, H-14), 7.59 (t, ^3^J = 7 Hz, 2H, H-13), 7.95 (d, ^3^J = 9 Hz, 1H, H-8), 8.13 (dd, ^3^J = 9 Hz, ^4^J = 2 Hz, 1H, H-7), 8.30 (d, ^3^J = 7 Hz, 2H, H-12), 8.52 (s, 1H, H-3), 9.14 (d, ^4^J = 2 Hz, 1H, H-5), 14.03 (bs, 1H, OH).

^13^C NMR (150.9 MHz, DMSO-d_6_, δ (ppm)): 94.8 (C-6), 120.2 (CH-3), 125.1 (C-10), 127.2 (CH-12), 129.0 (CH-13), 130.2 (CH-14), 131.6 (CH-8), 133.9 (CH-5), 135.9 (C-4), 137.5 (C-11), 138.5 (CH-7), 147.3 (C-9), 156.4 (C-2), 167.1 (COOH).

HRMS (MALDI-TOF/TOF) *m*/*z*: [M + H]^+^ Calcd for C_16_H_11_INO_2_ 375.9834; found 375.9827.


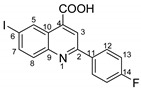


**2-(4-fluorophenyl)-6-iodoquinoline-4-carboxylic acid 4b:** crystallized from acetic acid; yellow powder; 82% yield; mp 131–132 °C; IR ATR ν(cm^−1^): 3649, 3272, 3066, 2982, 2971, 2884, 1684, 1653, 1594, 1540, 1507, 1481, 1373, 1328, 1259, 1233, 1155, 1088, 1020, 957, 940, 828, 677, 585, 507.

^1^H NMR (600.1 MHz, DMSO-d_6_, δ (ppm)): 7.40 (t, ^3^J_H,H_ = ^3^J_H,F_ = 9 Hz, 2H, H-13), 7.92 (d, ^3^J = 9 Hz, 1H,H-8), 8.11 (dd, ^3^J = 9 Hz, ^4^J = 2 Hz, 1H, H-7), 8.36 (dd, ^3^J_H,H_ = 9 Hz, ^4^J_H,F_ = 5 Hz, 2H, H-12), 8.50 (s, 1H, H-3), 9.12 (d, ^4^J = 2 Hz, 1H, H-5), 14.14 (bs, 1H, OH).

^13^C NMR (150.9 MHz, DMSO-d_6_, δ (ppm)): 94.8 (C-6), 115.9 (d, ^2^J_C,F_ = 22 Hz, CH-13), 119.9 (CH-3), 125.0 (C-10), 129.6 (d, ^3^J_C,F_ = 8 Hz, CH-12), 131.5 (CH-8), 133.9 (CH-5), 134.0 (d, ^4^J_C,F_ = 2 Hz, C-11), 135.9 (C-4), 138.5 (C-11), 138.5 (CH-7), 147.2 (C-9), 155.3 (C-2), 163.5 (d, ^1^J_C,F_ = 246 Hz, C-14), 167.1 (COOH).

HRMS (MALDI-TOF/TOF) *m*/*z*: [M + H]^+^ Calcd for C_16_H_10_FINO_2_ 393.9740; found 393.9723.


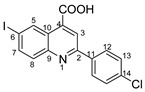


**2-(4-chlorophenyl)-6-iodoquinoline-4-carboxylic acid 4c:** yellow powder; 79% yield; mp 171–172 °C; IR ATR ν(cm^−1^): 3370, 3113, 3051, 1732, 1660, 1581, 1529, 1483, 1319, 1259, 1222, 1130, 1089, 1047, 1012, 943, 858, 808, 796, 549, 505. 

^1^H NMR (400.1 MHz, DMSO-d_6_, δ (ppm)): 7.63 (d, ^3^J = 9 Hz, 2H, H-13), 7.92 (d, ^3^J = 9 Hz, 1H, H-8), 8.11 (dd, ^3^J = 9 Hz, ^4^J = 2 Hz, 1H, H-7), 8.32 (d, ^3^J = 9 Hz, 2H, H-12), 8.51 (s, 1H, H-3), 9.12 (d, ^4^J = 2 Hz, 1H, H-5), 13.81 (bs, 1H, OH).

^13^C NMR (100.6 MHz, DMSO-d_6_, δ (ppm)): 95.1 (C-6), 120.0 (CH-3), 125.2 (C-10), 129.0 (CH-12 and CH-13), 131.5 (CH-8), 133.9 (CH-5), 135.1 (C-14), 136.0 (C-11), 136.2 (C-4), 138.6 (CH-7), 147.2 (C-9), 155.1(C-2), 167.0 (COOH).

HRMS (MALDI-TOF/TOF) *m*/*z*: [M + H]^+^ Calcd for C_16_H_10_ClINO_2_ 409.9444; found 409.9428.


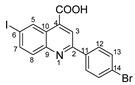


**2-(4-bromophenyl)-6-iodoquinoline-4-carboxylic acid 4d:** crystallized from acetic acid; yellow crystals; 69% yield; mp 268–270 °C; IR ATR ν(cm^−1^): 3676, 3649, 3630, 3103, 2989, 2897, 2496, 2365, 1685, 1581, 1539, 1477, 1402, 1280, 1251, 1188, 1072, 1010, 887, 829, 788, 727, 665, 619, 565, 482, 445, 414. 

^1^H NMR (400.1 MHz, DMSO-d_6_, δ (ppm)): 7.77 (d, ^3^J = 9 Hz, 2H, H-13), 7.93 (d, ^3^J = 9 Hz, 1H, H-8), 8.12 (dd, ^3^J = 9 Hz, ^4^J = 2 Hz, 1H, H-7), 8.26 (d, ^3^J = 9 Hz, 2H, H-12), 8.51 (s, 1H, H-3), 9.13 (d, ^4^J = 2 Hz, 1H, H-5), 14.01 (bs, 1H, OH).

^13^C NMR (100.6 MHz, DMSO-d_6_, δ (ppm)): 95.1 (C-6), 119.9 (CH-3), 124.0 (C-14), 125.2 (C-10), 129.3 (CH-12), 131.5 (CH-8), 131.9 (CH-13), 133.9 (CH-5), 136.0 (C-11), 136.6 (C-4), 138.6 (CH-7), 147.2 (C-9), 155.2 (C-2), 166.9 (COOH).

HRMS (MALDI-TOF/TOF) *m*/*z*: [M + H]^+^ Calcd for C_16_H_10_BrINO_2_ 453.8939; found 453.8927.


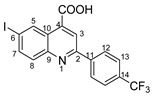


**6-iodo-2-(4-(trifluoromethyl)phenyl)quinoline-4-carboxylic acid 4e:** crystallized from acetic acid; yellow powder; 62% yield; mp 185–186 °C; IR ATR ν(cm^−1^): 3334, 3320, 2361, 1698, 1652, 1582, 1522, 1488, 1321, 1164, 1109, 1064, 1015, 1006, 912, 854, 829, 812, 773, 601.

^1^H NMR (600.1 MHz, DMSO-d_6_, δ (ppm)): 7.92 (d, ^3^J = 8 Hz, 2H, H-13), 7.95 (d, ^3^J = 9 Hz, 1H, H-8), 8.14 (dd, ^3^J = 9 Hz, ^4^J = 2 Hz, 1H, H-7), 8.50 (d, ^3^J = 8 Hz, 2H, H-12), 8.57 (s, 1H, H-3), 9.15 (d, ^4^J = 2 Hz, 1H, H-5), 14.20 (bs, 1H, OH).

^13^C NMR (150.9 MHz, DMSO-d_6_, δ (ppm)): 95.6 (C-6), 120.3 (CH-3), 124.2 (q, ^1^J_C,F_ = 272 Hz, CF_3_), 125.4 (C-10), 125.8 (q, ^3^J_C,F_ = 3 Hz, CH-13), 128.0 (CH-12), 130.0 (q, ^2^J_C,F_ = 32 Hz, C-14), 131.7 (CH-8), 134.0 (CH-5), 136.6 (C-4), 138.7 (CH-7), 141.2 (C-11), 147.2 (C-9), 154.8 (C-2), 166.9 (COOH).

HRMS (MALDI-TOF/TOF) *m*/*z*: [M + H]^+^ Calcd for C_17_H_10_F_3_INO_2_ 443.9708; found 443.9701.


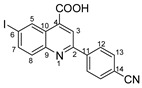


**2-(4-cyanophenyl)-6-iodoquinoline-4-carboxylic acid 4f:** crystallized from acetic acid; yellow powder; 90% yield; mp 287–289 °C; IR ATR ν(cm^−1^): 3649, 3080, 2982, 2971, 2884, 2245, 2229, 1720, 1700, 1653, 1588, 1540, 1485, 1382, 1333, 1277, 1225, 1172, 1055, 1019, 953, 898, 886, 842, 825, 788, 673, 641, 569, 549.

^1^H NMR (400.1 MHz, DMSO-d_6_, δ (ppm)): 7.91 (d, ^3^J = 9 Hz, 1H, H-8), 8.01 (d, ^3^J = 9 Hz, 2H, H-13), 8.12 (dd, ^3^J = 9 Hz, ^4^J = 2 Hz, 1H, H-7), 8.45 (d, ^3^J = 9 Hz, 2H, H-12), 8.54 (s, 1H, H-3), 9.12 (d, ^4^J = 2 Hz, 1H, H-5), 13.00 (bs, 1H, OH).

^13^C NMR (100.6 MHz, DMSO-d_6_, δ (ppm)): 95.9 (C-6), 112.4 (C-14), 118.6 (CN), 120.4 (CH-3), 125.4 (C-10), 128.0 (CH-12), 131.7 (CH-8), 132.9 (CH-13), 134.0 (CH-5), 136.2 (C-4), 138.8 (CH-7), 141.5 (C-11), 147.2 (C-9), 154.4 (C-2), 166.9 (COOH).

HRMS (MALDI-TOF/TOF) *m*/*z*: [M + H]^+^ Calcd for C_17_H_10_IN_2_O_2_ 400.9787; found 400.9767.


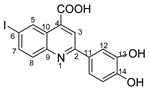


**2-(3,4-dihydroxyphenyl)-6-iodoquinoline-4-carboxylic acid 4i**: crystallized from acetic acid; orange powder; 89% yield; mp 309–311 °C; IR ATR ν(cm^−1^): 3650, 3271, 2982, 2971, 2884, 1714, 1653, 1598, 1575, 1540, 1436, 1395, 1375, 1329, 1295, 1260, 1223, 1169, 1153, 1081, 1015, 949, 890, 878, 863, 808, 787, 756, 711, 611, 591, 536, 521, 501.

^1^H NMR (400.1 MHz, DMSO-d_6_, δ (ppm)): 6.91 (d, ^3^J = 8 Hz, 1H, H-15), 7.60 (dd, ^3^J = 8 Hz, ^4^J = 2 Hz, 1H, H-16), 7.78 (d, ^4^J = 2 Hz, 1H, H-12), 7.84 (d, ^3^J = 9 Hz, 1H, H-8), 8.05 (dd, ^3^J = 9 Hz, ^4^J = 2 Hz, 1H, H-7), 8.37 (s, 1H, H-3), 9.09 (d, ^4^J = 1 Hz, 1H, H-5), 9.31 (bs, 1H, OH), 9.50 (bs, 1H, OH), 13.00 (bs, 1H, OH).

^13^C NMR (100.6 MHz, DMSO-d_6_, δ (ppm)): 93.6 (C-6), 114.2 (CH-12), 115.9 (CH-15), 119.2 (CH-16), 119.7 (CH-3), 124.8 (C-10), 128.8 (C-11), 131.3 (CH-8), 133.9 (CH-5), 135.2 (C-4), 138.3 (CH-7), 145.8 (C-13), 147.4 (C-9), 148.1 (C-14), 156.4 (C-2), 167.2 (COOH).

HRMS (MALDI-TOF/TOF) *m*/*z*: [M + H]^+^ Calcd for C_16_H_11_INO_4_ 407.9733; found 407.9745.


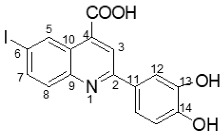


**6-iodo-2-(4-methoxyphenyl)quinoline-4-carboxylic acid 4k**: crystallized from acetic acid; yellow powder; 69% yield; mp 242–243 °C; IR ATR ν(cm^−1^): 3016, 2970, 1740, 1717, 1540, 1482, 1419, 1365, 1229, 1218, 1170, 1025, 844, 819, 787, 750, 681, 644, 511.

^1^H NMR (400.1 MHz, DMSO-d_6_, δ (ppm)): 3.86 (s, 3H, OCH_3_), 7.12 (d, ^3^J = 9 Hz, 2H, H-13), 7.88 (d, ^3^J = 9 Hz, 1H, H-8), 8.08 (dd, ^3^J = 9 Hz, ^4^J = 2 Hz, 1H, H-7), 8.27 (d, ^3^J = 9 Hz, 2H, H-12), 8.46 (s, 1H, H-3), 9.09 (d, ^4^J = 2 Hz, 1H, H-5), 14.05 (bs, 1H, OH).

^13^C NMR (100.6 MHz, DMSO-d_6_, δ (ppm)): 55.3 (OCH_3_), 94.0 (C-6), 114.4 (CH-13), 119.7 (CH-3), 124.8 (C-10), 128.8 (CH-12), 129.9 (C-11), 130.7 (CH-8), 133.9 (CH-5), 135.6 (C-4), 138.4 (CH-7), 147.3 (C-9), 155.2 (C-2), 161.1 (C-14), 167.01 (COOH).

HRMS (MALDI-TOF/TOF) *m*/*z*: [M + H]^+^ Calcd for C_17_H_10_IN_2_O_2_ 405.9940; found 405.9960.


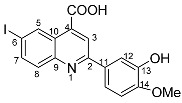


**2-(3-hydroxy-4-methoxyphenyl)-6-iodoquinoline-4-carboxylic acid 4s**: crystallized from acetic acid; orange powder; 85% yield; mp 292–295 °C; IR ATR ν(cm^−1^): 3648, 3090, 2982, 2970, 2883, 1717, 1638, 1598, 1571, 1516, 1396, 1380, 1304, 1263, 1222, 1146, 1077, 1019, 952, 867, 809, 797, 770, 581, 524, 509.

^1^H NMR (400.1 MHz, DMSO-d_6_, δ (ppm)): 3.87 (s, 3H, CH_3_), 7.09 (d, ^3^J = 8 Hz, 1H, H-15), 7.72 (dd, ^3^J = 8 Hz, ^4^J = 2 Hz, 1H, H-16), 7.81 (d, ^4^J = 2 Hz, 1H, H-12), 7.87 (d, ^3^J = 9 Hz, 1H, H-8), 8.08 (dd, ^3^J = 9 Hz, ^4^J = 2 Hz, 1H, H-7), 8.41 (s, 1H, H-3), 9.11 (d, ^4^J = 1 Hz, 1H, H-5), 9.36 (bs, 1H, OH), 13.97 (bs, 1H, OH).

^13^C NMR (100.6 MHz, DMSO-d_6_, δ (ppm)): 55.6 (OCH_3_), 93.9 (C-6), 112.1 (CH-12), 113.9 (CH-15), 118.9 (CH-16), 119.8 (CH-3), 124.9 (C-10), 130.2 (C-11), 131.3 (CH-8), 133.9 (CH-5), 135.4 (C-4), 138.4 (CH-7), 146.9 (C-13), 147.3 (C-9), 149.9 (C-14), 156.1 (C-2), 167.1 (COOH).

HRMS (MALDI-TOF/TOF) *m*/*z*: [M + H]^+^ Calcd for C_17_H_13_INO_4_ 421.9889; found 421.9896.


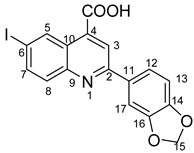


**2-(benzo[d][1,3]dioxol-5-yl)-6-iodoquinoline-4-carboxylic acid 4t**: crystallized from acetic acid; brown powder; 77% yield; mp 205–206 °C; IR ATR ν(cm^−1^): 3660, 2978, 2889, 1744, 1604, 1580, 1505, 1452, 1372,1352, 1239, 1152, 1114, 1033, 941, 867, 825, 809, 603, 519.

^1^H NMR (400.1 MHz, DMSO-d_6_, δ (ppm)): 6.15 (s, 2H, CH_2_), 7.09 (d, ^3^J = 8 Hz, 1H, H-13), 7.83–7.86 (m, 2H, H-12 and H-17), 7.88 (d, ^3^J = 9 Hz, 1H, H-8), 8.08 (dd, ^3^J = 9 Hz, ^4^J = 2 Hz, 1H, H-7), 8.44 (s, 1H, H-3), 9.08 (d, ^4^J = 1 Hz, 1H, H-5), 9.36 (bs, 1H, OH), 13.06 (bs, 1H, OH).

^13^C NMR (100.6 MHz, DMSO-d_6_, δ (ppm)): 94.2 (C-6), 101.6 (CH_2_), 106.9 (CH-17), 108.6 (CH-13), 119.8 (CH-3), 122.0 (CH-12), 124.9 (C-10), 131.4 (C-11), 131.8 (CH-8), 133.8 (CH-5), 135.8 (C-4), 138.4 (CH-7), 147.1 (C-9), 148.2 (C-14), 149.2 (C-16), 155.7 (C-2), 167.1 (COOH).

HRMS (MALDI-TOF/TOF) *m*/*z*: [M + H]^+^ Calcd for C_17_H_11_INO_4_ 419.9733; found 419.9708.


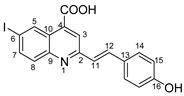


**(*E*)-2-(4-hydroxystyryl)-6-iodoquinoline-4-carboxylic acid 5h**: crystallized from acetic acid; yellow powder; 57% yield; mp 221–223 °C; IR ATR ν(cm^−1^): 3314, 3024, 2970, 2953, 2922, 2853, 1740, 1666, 1646, 1582, 1534, 1487, 1415, 1386, 1231, 1217, 1162, 1063, 1008, 916, 828, 814, 792, 777, 742, 633, 620, 543.

^1^H NMR (400.1 MHz, DMSO-d_6_, δ (ppm)): 6.84 (d, ^3^J = 8 Hz, 2H, H-15), 7.33 (d, ^3^J = 16 Hz, 1H, H-11), 7.62 (d, ^3^J = 8 Hz, 2H, H-14), 7.82 (d, ^3^J = 9 Hz, 1H, H-8), 7.84 (d, ^3^J = 16 Hz, 1H, H-12), 8.05 (dd, ^3^J = 9 Hz, ^4^J = 2 Hz, 1H, H-7), 8.24 (s, 1H, H-3), 9.08 (d, ^4^J = 1 Hz, 1H, H-5), 9.85 (bs, 1H, OH), 14.04 (bs, 1H, OH).

^13^C NMR (100.6 MHz, DMSO-d_6_, δ (ppm)): 93.6 (C-6), 115.7 (CH-15), 121.6 (CH-3), 124.2 (CH-11), 124.9 (C-10), 127.0 (C-13), 129.2 (CH-14), 130.9 (CH-8), 133.9 (CH-5), 134.9 (C-4), 135.6 (CH-12), 138.2 (CH-7), 147.4 (C-9), 156.4 (C-2), 158.6 (C-16), 167.2 (COOH).

HRMS (MALDI-TOF/TOF) *m*/*z*: [M + H]^+^ Calcd for C_18_H_13_INO_3_ 417.9940; found 417.9931.


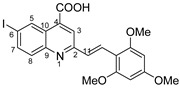


**(*E*)-6-iodo-2-(2,4,6-trimethoxystyryl)quinoline-4-carboxylic acid 5n**: crystallized from acetic acid; yellow powder; 47% yield; mp 328–329 °C; IR ATR ν(cm^−1^): 3649, 2982, 2971, 2891, 1560, 1380, 1253, 1154, 1073, 967, 809, 519. 

^1^H NMR (400.1 MHz, DMSO-d_6_, δ (ppm)): 3.87 (s, 3H, OCH_3_), 3.94 (s, 6H, 2xOCH_3_), 6.35 (s, 2H, H-15), 7.67 (d, ^3^J = 16 Hz, 1H, H-11), 7.82 (d, ^3^J = 9 Hz, 1H, H-8), 8.06 (dd, ^3^J = 9 Hz, ^4^J = 2 Hz, 1H, H-7), 8.11 (s, 1H, H-3), 8.14 (d, ^3^J = 16 Hz, 1H, H-12), 9.07 (d, ^4^J = 1 Hz, 1H, H-5), 13.08 (bs, 1H, OH).

^13^C NMR (100.6 MHz, DMSO-d_6_, δ (ppm)): 55.4 (OCH_3_), 55.9 (2xOCH_3_), 91.0 (CH-15), 93.3 (C-6), 105.9 (C-13), 121.9 (CH-3), 124.9 (C-10), 126.6 (CH-12), 126.9 (CH-11), 130.9 (CH-8), 133.9 (CH-5), 134.7 (C-4), 138.2 (CH-7), 147.4 (C-9), 157.5 (C-2), 160.2 (C-14), 161.7 (C-16), 167.1 (COOH). 

HRMS (MALDI-TOF/TOF) *m*/*z*: [M + H]^+^ Calcd for C_21_H_19_INO_5_ 492.0308; found 492.0335.


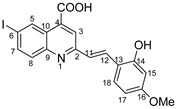


**(*E*)-2-(2-hydroxy-4-methoxystyryl)-6-iodoquinoline-4-carboxylic acid 5o**: crystallized from acetic acid; yellow powder; 59% yield; mp 226–227 °C; IR ATR ν(cm^−1^): 3649, 2982, 2970, 2889, 1700, 1576, 1558, 1382, 1255, 1223, 1147, 1074, 968, 95, 809, 737, 668.

^1^H NMR (400.1 MHz, DMSO-d_6_, δ (ppm)): 3.76 (s, 3H, OCH_3_), 6.50–6.51 (m, 2H, H-15and H-17), 7.41 (d, ^3^J = 16 Hz, 1H, H-11), 7.66 (d, ^3^J = 9 Hz, 1H, H-18), 7.82 (d, ^3^J = 9 Hz, 1H, H-8), 8.05 (dd, ^3^J = 9 Hz, ^4^J = 2 Hz, 1H, H-7), 8.05 (d, ^3^J = 16 Hz, 1H, H-12), 8.17 (s, 1H, H-3), 9.08 (d, ^4^J = 2 Hz, 1H, H-5), 10.20 (bs, 1H, OH), 13.74 (bs, 1H, OH).

^13^C NMR (150.9 MHz, DMSO-d_6_, δ (ppm)): 55.0 (OCH_3_), 93.5 (C-6), 101.2 (CH-15), 105.9 (CH-17), 115.8 (C-13), 121.7 (CH-3), 124.4 (CH-11), 124.9 (C-10), 128.8 (CH-18), 130.8 (CH-12), 131.1 (CH-8), 133.9 (CH-5), 134.7 (C-4), 138.2 (CH-7), 147.5 (C-9), 156.8 (C-2), 157.4 (C-13), 161.1 (C-16), 167.1 (COOH).

HRMS (MALDI-TOF/TOF) *m*/*z*: [M + H]^+^ Calcd for C_19_H_15_INO_4_ 448.0046; found 448.0029.


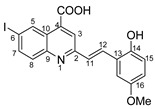


**(*E*)-2-(2-hydroxy-5-methoxystyryl)-6-iodoquinoline-4-carboxylic acid 5p**: crystallized from acetic acid; brown powder; 74% yield; mp 324–325 °C; IR ATR ν(cm^−1^): 3648, 2981, 2971, 2891, 1653, 1593, 1559, 1383, 1262, 1225, 1150, 1075, 1017, 966, 949, 865, 807, 579.

^1^H NMR (600.1 MHz, DMSO-d_6_, δ (ppm)): 3.77 (s, 3H,OCH_3_), 6.82 (dd, ^3^J = 9 Hz, ^4^J = 3 Hz, 1H, H-16), 6.87 (d, ^3^J = 9 Hz, 1H, H-15), 7.30 (d, ^3^J = 3 Hz, 1H, H-18), 7.58 (d, ^3^J = 16 Hz, 1H, H-11), 7.85 (d, ^3^J = 9 Hz, 1H, H-8), 8.07 (dd, ^3^J = 9 Hz, ^4^J = 2 Hz, 1H, H-7), 8.10 (d, ^3^J = 16 Hz, 1H, H-12), 8.21 (s, 1H, H-3), 9.12 (d, ^4^J = 2 Hz, 1H, H-5), 9.62 (bs, 1H, OH), 14.04 (bs, 1H, OH).

^13^C NMR (150.9 MHz, DMSO-d_6_, δ (ppm)): 55.4 (OCH_3_), 93.9 (C-6), 110.9 (CH-18), 116.9 (CH-16), 117.0 (CH-15), 122.0 (CH-3), 122.9 (C-13), 125.1 (C-10), 127.0 (CH-11), 130.6 (CH-12), 131.1 (CH-8), 134.0 (C-4), 134.8 (CH-5), 138.3 (CH-7), 147.5(C-9), 150.1 (C-14), 152.3 (C-17), 156.4 (C-2), 167.0 (COOH).

HRMS (MALDI-TOF/TOF) *m*/*z*: [M + H]^+^ Calcd for C_19_H_15_INO_4_ 448.0046; found 448.0032.


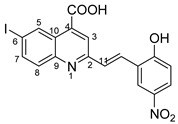


**(*E*)-2-(2-hydroxy-5-nitrostyryl)-6-iodoquinoline-4-carboxylic acid 5r**: crystallized from acetic acid; yellow powder; 88% yield; mp 328–330 °C; IR ATR ν(cm^−1^): 3650, 3114, 3084, 2982, 2971, 2883, 1700, 1683, 1654, 1594, 1559, 1483, 1381, 1339, 1266, 1147, 969, 943, 826, 748, 691, 628.

^1^H NMR (600.1 MHz, DMSO-d_6_, δ (ppm)): 7.13 (d, ^3^J = 9 Hz, 1H, H-15), 7.81 (d, ^3^J = 16 Hz, 1H, H-11), 7.88 (d, ^3^J = 9 Hz, 1H, H-8), 8.09 (dd, ^3^J = 9 Hz, ^4^J = 2 Hz, 1H, H-7), 8.13 (dd, ^3^J = 9 Hz, ^4^J = 3 Hz, 1H, H-16), 8.13 (d, ^3^J = 16 Hz, 1H, H-12), 8.30 (s, 1H, H-3), 8.64 (d, ^4^J = 3 Hz, 1H, H-18), 9.13 (d, ^4^J = 2 Hz, 1H, H-5), 11.76 (bs, 1H, OH), 14.06 (bs, 1H, OH).

^13^C NMR (150.9 MHz, DMSO-d_6_, δ (ppm)): 94.5 (C-6), 116.4 (CH-15), 122.5 (CH-3), 123.5 (C-13), 123.7 (CH-18), 125.3 (C-10), 125.5 (CH-16), 128.6 (CH-12), 129.8 (CH-11), 131.2 (CH-8), 133.9 (CH-5), 135.0 (C-4), 138.4 (CH-7), 140.0 (C-17), 147.4 (C-9), 155.8 (C-2), 161.8 (C-14), 167.0 (COOH). 

HRMS (MALDI-TOF/TOF) *m*/*z*: [M + H]^+^ Calcd for C_18_H_12_IN_2_O_5_ 462.9791; found 462.9799.


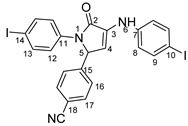


**4-(1-(4-iodophenyl)-4-((4-iodophenyl)amino)-5-oxo-2,5-dihydro-1H-pyrrole-2-yl)benzonitrile 6f**: [13]


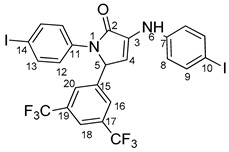


**5-(3,5-bis(trifluoromethyl)phenyl)-1-(4-iodophenyl)-3-((4-iodophenyl)amino)-1H-pyrrole-2(5H)-one 6g**: [13]


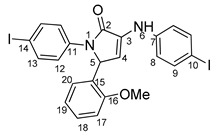


**1-(4-iodophenyl)-3-((4-iodophenyl)amino)-5-(2-methoxyphenyl)-1H-pyrrole-2(5H)-one 6j**: [13]


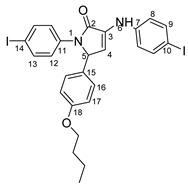


**5-(4-butoxyphenyl)-1-(4-iodophenyl)-3-((4-iodophenyl)amino)-1H-pyrrole-2(5H)-one 6l**: crystallized from acetic acid; black powder; 53% yield; mp 229–230 °C; IR ATR ν(cm^−1^): 3117, 3077, 3027, 2944, 1738, 1595, 1484, 1434, 1369, 1284, 1230, 1217, 1174, 1143, 840, 827, 668, 628, 529.

^1^H NMR (400.1 MHz, DMSO-d_6_, δ (ppm)): 0.90 (t, ^3^J = 7 Hz, 3H,CH_3_), 1.39 (sextet, ^3^J = 7 Hz, 2H, CH_2_), 1.64 (quintet, ^3^J = 7 Hz, 2H,CH_2_), 3.88 (t, ^3^J = 7 Hz, 2H, OCH_2_), 5.98 (d, ^3^J = 2 Hz, 1H, H-5), 6.33 (d, ^3^J = 2 Hz, 1H, H-4), 6.83 (d, ^3^J = 8 Hz, 2H, H-17), 7.14 (d, ^3^J = 8 Hz, 4H, H-8 and H-16), 7.45 (d, ^3^J = 9 Hz, 2H, H-12), 7.54 (d, ^3^J = 9 Hz, 2H, H-9), 7.66 (d, ^3^J = 9 Hz, 2H, H-13), 8.32 (s, 1H, NH).

^13^C NMR (100.6 MHz, DMSO-d_6_, δ (ppm)): 13.6 (CH_3_), 18.7 (CH_2_-21), 30.6 (CH_2_-20), 61.7 (CH-5), 67.0 (CH_2_-19), 82.4 (C-10), 88.9 (C-14), 111.3 (CH-4), 114.6 (CH-17), 119.1 (CH-8), 123.5 (CH-12), 128.0 (CH-16), 128.7 (C-15), 131.3 (C-3), 136.8 (C-11), 137.3 (CH-13), 137.4 (CH-9), 141.8 (C-7), 158.3 (C-18), 166.2 (CO-2).

HRMS (MALDI-TOF/TOF) *m*/*z*: [M + H]^+^ Calcd for C_26_H_25_I_2_N_2_O_2_ 651.0005; found 651.0027.


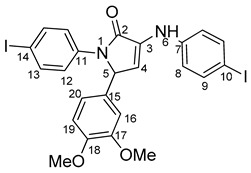


**5-(3,4-dimethoxyphenyl)-1-(4-iodophenyl)-3-((4-iodophenyl)amino)-1H-pyrrole-2(5H)-one 6m**: [13]


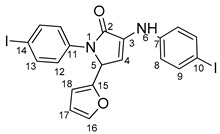


**5-(furan-2-yl)-1-(4-iodophenyl)- 3-((4-iodophenyl)amino)-1H-pyrrole-2(5H)-one 6u**: [13]


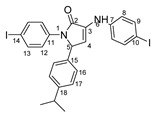


**1-(4-iodophenyl)-3-((4-iodophenyl)amino)-5-(4-isopropylphenyl)-1H-pyrrole-2(5H)-one 6v**: crystallized from acetic acid; black powder; 53% yield; mp 197–200 °C; IR ATR ν(cm^−1^): 3649, 3315, 2982, 2971, 2883, 1666, 1648, 1584, 1533, 1487, 1456, 1417, 1390, 1264, 1162, 1060, 1008, 957, 813, 792, 776, 638, 560, 507.

^1^H NMR (600.1 MHz, DMSO-d_6_, δ (ppm)): 1.13 (d, ^3^J = 7 Hz, 3H, CH_3_), 1.14 (d, ^3^J = 7 Hz, 3H, CH_3_), 2.81 (septet, ^3^J = 7 Hz, 1H, CH), 6.02 (d, ^3^J = 3 Hz, 1H, H-5), 6.36 (d, ^3^J = 3 Hz, 1H, H-4), 7.14 (d, ^3^J = 9 Hz, 2H, H-8), 7.17 (bs, 4H, H-16 and H-17), 7.47 (d, ^3^J = 9 Hz, 2H, H-12), 7.53 (d, ^3^J = 9 Hz, 2H, H-9), 7.68 (d, ^3^J = 9 Hz, 2H, H-13), 8.31 (s, 1H, NH).

^13^C NMR (150.9 MHz, DMSO-d_6_, δ (ppm)): 23.7 (2xCH_3_), 32.9 (CH), 61.9 (CH-5), 82.5 (C-10), 88.9 (C-14), 111.3 (CH-4), 119.1 (CH-8), 123.4 (CH-12), 127.7 and 127.8 (CH-16 and CH-17), 131.2 (C-3), 134.7 (C-15), 136.9 (C-11), 137.4 (CH-9 and CH-13), 141.8 (C-7), 147.9 (C-18), 166.3 (CO-2).

HRMS (MALDI-TOF/TOF) *m*/*z*: [M + H]^+^ Calcd for C_25_H_23_I_2_N_2_O 620.9909; found 620.9929.


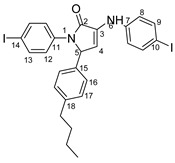


**5-(4-butylphenyl)-1-(4-iodophenyl)-3-((4-iodophenyl)amino)-1H-pyrrole-2(5H)-one 6x**: crystallized from acetic acid; black powder; 53% yield; mp 211–212 °C; IR ATR ν(cm^−1^): 3313, 3024, 2956, 2927, 2854, 1739, 1665, 1646, 1585, 1535, 1487, 1416, 1388, 1229, 1218, 1164, 1063, 1009, 914, 828, 813, 793, 776, 741, 636, 621 544.

^1^H NMR (600.1 MHz, DMSO-d_6_, δ (ppm)): 0.86 (t, ^3^J = 7 Hz, 3H, CH_3_), 1.26 (sextet, ^3^J = 7 Hz, 2H, CH_2_), 1.48 (quintet, ^3^J = 7 Hz, 2H, CH_2_), 2.47–2.49 (m, 2H, CH_2_ overlapped with DMSO), 6.00 (d, ^3^J = 2 Hz, 1H, H-5), 6.36 (d, ^3^J = 2 Hz, 1H, H-4), 7.09–7.15 (m, 6H, H-8, H-16, H-17), 7.45 (d, ^3^J = 9 Hz, 2H, H-12), 7.54 (d, ^3^J = 9 Hz, 2H, H-9), 7.66 (d, ^3^J = 9 Hz, 2H, H-13), 8.32 (s, 1H, NH).

^13^C NMR (100.6 MHz, DMSO-d_6_, δ (ppm)): 13.7 (CH_3_), 21.7 (CH_2_-21), 32.9 (CH_2_-20), 34.4 (CH-19), 62.0 (CH-5), 82.5 (C-10), 88.9 (C-14), 111.2 (CH-4), 119.1 (CH-8), 123.4 (CH-12), 126.6 (CH-16), 128.7 (CH-17), 131.2 (C-3), 134.5 (C-15), 136.8 (C-11), 137.3 (CH-13), 137.4 (CH-9), 141.8 (C-7), 142.0 (C-18), 166.3 (CO-2).

HRMS (MALDI-TOF/TOF) *m*/*z*: [M + H]^+^ Calcd for C_26_H_25_I_2_N_2_O 635.0056; found 635.0032.


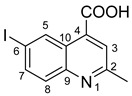


**6-iodo-2-methylquinoline-4-carboxylic acid 7**: crystallized from acetic acid; brown powder; 37% yield; mp 289–290 °C; IR ATR ν(cm^−1^): 3656, 3083, 2979, 2887, 1714, 1603, 1579, 1504, 1454, 1377, 1351, 1240, 1152, 1113, 1032, 937, 867, 810, 604, 519. 

^1^H NMR (600.1 MHz, DMSO-d_6_, δ (ppm)): 2.71 (s, 3H, CH_3_), 7.79 (d, ^3^J = 9 Hz, 1H, H-8), 7.89 (s, 1H, H-3), 8.05 (dd, ^3^J = 9 Hz, ^4^J = 2 Hz, 1H, H-7), 9.10 (d, ^4^J = 2 Hz, 1H, H-5), 14.00 (bs, 1H, OH).

^13^C NMR (150.9 MHz, DMSO-d_6_, δ (ppm)): 24.7 (CH_3_), 93.7 (C-6), 123.8 (CH-3), 124.5 (C-10), 130.8 (CH-8), 133.9 (CH-5), 134.4 (C-4), 137.9 (CH-7), 147.0 (C-9), 159.6 (C-2), 167.1 (COOH).

HRMS (MALDI-TOF/TOF) *m*/*z*: [M + H]^+^ Calcd for C_11_H_9_INO_2_ 313.9678; found 313.9664.


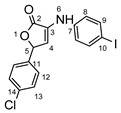


**5-(4-chlorophenyl)-3-((4-iodophenyl)amino)furan-2(5H)-one 8c**: crystallized from acetic acid; yellow powder; 27% yield; mp 161–162 °C; IR ATR ν(cm^−1^): 3460, 3304, 3020, 2968, 1741, 1575, 1535, 1444, 1371, 1222, 1092, 912, 802, 528.

^1^H NMR (400.1 MHz, DMSO-d_6_, δ (ppm)): 6.23 (d, ^3^J = 2 Hz, 1H, H-5), 6.82 (d, ^3^J = 2 Hz, 1H, H-4), 7.15 (d, ^3^J = 9 Hz, 2H, H-8), 7.42 (d, ^3^J = 9 Hz, 2H, H-12), 7.50 (d, ^3^J = 9 Hz, 2H, H-13), 7.57 (d, ^3^J = 9 Hz, 2H, H-9), 8.59 (s, 1H,NH).

HRMS (MALDI-TOF/TOF) *m*/*z*: [M + H]^+^ Calcd for C_16_H_12_ClINO_2_ 411.9601, found 411.9584.


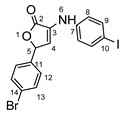


**5-(4-bromophenyl)-3-((4-iodophenyl)amino)furan-2(5H)-one 8d**: crystallized from acetic acid; brown powder; 31% yield; mp 182–184 °C; IR ATR ν(cm^−1^): 3463, 3071, 3031, 2942, 2360, 2245, 1740, 1722, 1588, 1366, 1226, 1218, 1205, 1173, 899, 823, 787, 674, 640, 569, 544, 519.

^1^H NMR (400.1 MHz, DMSO-d_6_, δ (ppm)): 6.21 (d, ^3^J = 2 Hz, 1H, H-5), 6.82 (d, ^3^J = 2 Hz, 1H, H-4), 7.14 (d, ^3^J = 9 Hz, 2H, H-8), 7.35 (d, ^3^J = 9 Hz, 2H, H-12), 7.57 (d, ^3^J = 9 Hz, 2H, H-13), 7.63 (d, ^3^J = 9 Hz, 2H, H-9), 8.60 (s, 1H, NH).

HRMS (MALDI-TOF/TOF) *m*/*z*: [M + H]^+^ Calcd for C_16_H_12_BrINO_2_ 455.9096, found 455.9078.


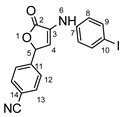


**4-(4-((4-iodophenyl)amino)-5-oxo-2,5-dihydrofuran-2-yl)benzonitrile 8f**: crystallized from acetic acid; brown powder; 29% yield; mp 184–185 °C; IR ATR ν(cm^−1^): 3463, 3071, 3031, 2942, 2360, 2245, 1740, 1722, 1588, 1366, 1226, 1218, 1205, 1173, 899, 823, 787, 674, 640, 569, 544, 519.

^1^H NMR (400.1 MHz, DMSO-d_6_, δ (ppm)): 6.32 (d, ^3^J = 2 Hz, 1H, H-5), 6.86 (d, ^3^J = 2 Hz, 1H, H-4), 7.14 (d, ^3^J = 9 Hz, 2H, H-8), 7.57 (d, ^3^J = 9 Hz, 2H, H-9), 7.60 (d, ^3^J = 9 Hz, 2H, H-12), 7.90 (d, ^3^J = 9 Hz, 2H, H-13), 8.63 (s, 1H, NH).

HRMS (MALDI-TOF/TOF) *m*/*z*: [M + H]^+^ Calcd for C_17_H_12_IN_2_O_2_ 402.9943; found 402.9942.

### 3.2. X-ray Crystallography

Single-crystal X-ray diffraction data for **4d** and **4e** were collected on an Oxford-Diffraction XCALIBUR Eos CCD diffractometer with graphite monochromated Mo Kα radiation, while **4b** and **4c** were collected on an XtaLAB Synergy, Dualflex, HyPix diffractometer using Cu Kα radiation. The unit cell determination and data integration were carried out using the CrysAlisPro package from Oxford Diffraction [29]. The multi-scan correction for absorption was applied. The structures were solved with the program SHELXT using the intrinsic phasing method and refined by the full-matrix least-squares method on *F*^2^ with SHELXL [30,31]. Olex2 was used as an interface to the SHELX programs [32]. Non-hydrogen atoms were refined anisotropically. Hydrogen atoms were added in idealized positions and refined using a riding model. Selected crystallographic data and structure refinement details are provided in Table S1 and the corresponding CIF files. The supplementary crystallographic data can be obtained free of charge via www.ccdc.cam.ac.uk (or from the Cambridge Crystallographic Data Centre, 12 Union Road, Cambridge CB2 1EZ, UK; fax: (+44) 1223–336-033; or deposit@ccdc.ca.ac.uk).

### 3.3. Powder X-ray

X-ray diffraction analysis was performed in a Rigaku Miniflex 600 diffractometer using CuKα emission in the angular range 2–50 °(2θ) with a scanning step of 0.01° and a recording rate of 2 °/min. 

#### 3.3.1. Antimicrobial Activity

##### Microbial Strains

The antimicrobial activity was performed on reference microbial strains belonging to Gram-positive bacteria (*Staphylococcus epidermidis* ATCC 12228), Gram-negative bacteria (*Klebsiella pneumoniae* ATCC 13368) and yeast (*Candida parapsilosis* ATCC 22019).

#### 3.3.2. Quantitative Evaluation of Antimicrobial Activity

In a 96-well plate, quantitative analysis was carried out using the serial binary microdilution technique in liquid media (Tryptone Soy Broth for bacteria and Sabouraud for yeast). Stock solutions of 10 mg/Ml in DMSO were prepared, with concentrations ranging from 5 to 0.016 mg/Ml. Simultaneously, under the same working circumstances, repeated dilutions with DMSO were performed to obtain the negative control. Each well received 10 µL of microbial solution adjusted to 1.5 × 10^8^ CFU/Ml from cultures cultivated for 18 to 24 h. MIC values were calculated both macroscopically, as the final concentration at which no microbial growth was seen, and spectrophotometrically. The absorbance of the microbial cultures was measured at 620 nm using a Molecular Devices FlexStation 3 UV-Vis spectrophotometer (San Jose, CA, USA). For each concentration, a distinct blank was created for each sample. The data were analyzed using the Prism GraphPad 9.0 software’s log (inhibitory) vs. response analysis function—Variable Slope (four parameters)—in order to generate the IC50 (the sample concentration that inhibits 50% microbiological viability from a microbial inoculum). To measure the minimal microbicidal concentrations (MMC), 5 uL of each well was spotted on a solid medium. The plates were incubated at 37 °C for 20–24 h. The MMC was defined as the last concentration at which no microbial colony development was detected.

#### 3.3.3. The Effect on Microbial Adhesion

Following the quantitative investigation of antimicrobial activity, microbial adherence was assessed using the slime technique following fixation with methanol and staining with crystal violet (0.1%). At 490 nm, the absorbance of biological material suspended in 33% acetic acid was determined.

#### 3.3.4. Statistical Analysis

The data were presented as means ± standard deviation (SD) (as established by duplicate analysis). GraphPad Prism 10v was used for statistical analysis. For comparison of the IC50, general structure and solvent utilized, data were analyzed using standard two-way ANOVA with a two-step increase for multiple comparisons (Tukey). The significance threshold was chosen at *p* < 0.05. 

## 4. Conclusions

This study presents the design and synthesis of a novel family of carboxy-quinolines containing an iodine atom. The compounds were produced utilizing a one-pot, two-component technique, with trifluoroacetic acid serving as an efficient catalyst. The applicable method is an advancement of the Doebner reaction, which involves beginning with aniline and withdrawing substituents to produce desired quinolines. This technology offers many benefits, including reduced reaction times, a simplified purification process, the use of cost-effective catalysts and high product yields.

A complete investigation regarding the influence of the starting aldehyde was made. The obtained compounds were characterized by physicochemical techniques (^1^H-NMR, ^13^C-NMR, MS, T-IR, melting point and monocrystal and powder X-ray in some cases) in order to confirm the structure and the synthesis procedure. 

The antibacterial activity of the new products was investigated and all tested series showed good activity against *S. epidermidis* and *C. parapsilosis.* The best antimicrobial activity was found for the compounds containing *p*-Br and Cl-phenyl substituents. The derivatives with *p*-CF_3_-phenyl and benzo[d][1,3]dioxole moieties also showed good activity against *S. epidermidis* and *C. parapsilosis.* These compounds inhibited the adhesion of *S. epidermidis* and *C. parapsilosis* cells. Due to the hydrophobic nature of these compounds, they can easily penetrate the *Stratum corneum*, thus increasing their bioavailability for skin infection. In this context, we propose to continue the research to find new strategies to prevent the formation of chronic wounds due to microbial biofilm formation. 

## Figures and Tables

**Table 1:** Compressive obtained structures depending on the starting materials.

1	4	5	6	7	8	1	4	5	6	7	8	1	4	5	6	7	8
**a**	x					**h**		x				**o**		x			
**b**	x					**i**	x					**p**		x			
**c**	x				x	**j**			x			**r**		x			
**d**	x				x	**k**	x			x		**s**	x				
**e**	x					**l**			x	x		**t**	x				
**f**	x		x		x	**m**			x	x		**u**			x		
**g**			x			**n**		x				**v**			x		
												**x**			x		

1: starting aldehyde material; 4: quinoline main structure; 5: ethylene-bridged quinolinic structure; 6: pyrrole-2-one structure; 7: methyl quinoline by-product; 8: furan-2-one by-product.

**Table 2:** Antimicrobial activity is expressed as MIC (mg/mL), MBC (mg/mL) and MBEC-(mg/mL).

	*S. epidermidis*			*K. pneumoniae*			*C. parapsilosis*		
	MIC (mg/mL)	MBC (mg/mL)	MICMA (mg/mL)	MIC (mg/mL)	MBC (mg/mL)	MICMA (mg/mL)	MIC (mg/mL)	MBC (mg/mL)	MICMA (mg/mL)
**4d**	0.063	≤0.625	0.063	1.25	5	1.25	0.3125	2.5	0.3125
**4a**	0.625	0.625	0.3125	5	5	0.625	0.625	2.5	0.625
**4b**	0.625	0.625	0.3125	1.25	5	1.25	0.625	1.25	0.625
**4e**	0.25	≤0.625	0.25	2.5	5	1.25	0.625	2.5	0.625
**4f**	0.625	0.625	0.625	1.25	5	1.25	0.625	1.25	0.625
**4i**	1.25	2.5	1.25	1.25	5	1.25	1.25	1.25	1.25
**4c**	0.063	≤0.625	0.063	1.25	5	1.25	0.3125	≤0.625	0.3125
**4s**	1.25	2.5	1.25	1.25	5	1.25	0.625	0.625	0.625
**4t**	0.25	≤0.625	0.25	1.25	5	1.25	0.3125	≤0.625	≤0.15625
**7**	0.625	1.25	1.25	1.25	5	1.25	1.25	1.25	0.625
**5r**	0.3125	5	0.3125	1.25	5	1.25	0.625	5	0.625
**5o**	1.25	2.5	2.5	1.25	5	1.25	10	5	1.25
**5p**	0.625	2.5	1.25	1.25	5	5	0.625	5	0.625
**DMSO**	2.5	2.5	2.5	1.25	5	1.25	1.25	1.25	1.25

MIC: minimum inhibitory concentration at which bacterial growth is completely inhibited; MBC: minimum concentration of drug that kills 99.9% of the test microorganisms in the original inoculum; MBEC: minimal concentration of the drug that kills 99.9% of biofilm-embedded bacteria; MICMA: minimum inhibitory concentrations for microbial adhesion.

## Data Availability

The data presented in this study are available in article and Supplementary Materials.

## Supplementary Materials

The following supporting information can be downloaded at: https://www.mdpi.com/article/10.3390/molecules29040772/s1, Figure S1.A. The 1H-NMR spectrum corresponding to compound **4c**, recorded in DMSO-d6, at 400.1 MHz; Figure S1.B. The 13C-NMR spectrum corresponding to compound **4c**, recorded in DMSO-d6, at 100.6 MHz; Figure S1.C. Detailed region of the H,C-HSQC spectrum corresponding to compound **4c**, showing the correlation signals for protonated carbons; Figure S1.D. The H,C-HMBC spectrum corresponding to compound **4c**, showing 2 or 3 bond correlation signals between protons and carbons, used mainly to assign quaternary carbons; Figure S1.E. MALDI-MS spectra of compound **4c**; Figure S1.F. FT-IR spectrum of compound **4c**; Figure S2.A. The 1H-NMR spectrum corresponding to compound **4t**, recorded in DMSO-d6, at 400.1 MHz; Figure S2.B. The 13C-NMR spectrum corresponding to compound **4t**, recorded in DMSO-d6, at 100.6 MHz; Figure S2.C. MALDI-MS spectrum of the compound **4t**; Figure S2.D. FT-IR spectrum of compound **4t**; Figure S3.A. The 1H-NMR spectrum corresponding to compound **8c**, recorded in DMSO-d6, at 400.1 MHz; Figure S3.B. MALDI-MS spectra of compound **8c**; Figure S3.C. FT-IR spectrum of compound **8c**; Figure S4.A. The 1H-NMR spectrum corresponding to compound **5n**, recorded in DMSO-d6, at 400.1 MHz; Figure S4.B. The 13C-NMR spectrum corresponding to compound **5n**, recorded in DMSO-d6, at 100.6 MHz; Figure S4.C. The H,C-HMBC spectrum corresponding to compound **5n**, recorded in DMSO-d6, showing correlation signals between vinylic protons and either quinoline’s CH-3 or benzaldehyde’s C-14 carbon atoms; Figure S4.D. Maldi-MS spectrum corresponding to compound **5n**; Figure S4.E. FT-IR spectrum of compound **5n**; Figure S5.A. The 1H-NMR spectrum corresponding to compound **7**, recorded in DMSO-d6, at 600.1 MHz; Figure S5.B. The 13C-NMR spectrum corresponding to compound **7**, recorded in DMSO-d6, at 150.9 MHz; Figure S5.C. Maldi-MS spectrum corresponding to compound **7**; Figure S5.D. FT-IR spectrum of compound **7**; Figure S6. View of the asymmetric unit (large atoms and bonds radii) showing its interaction with adjacent molecules for **4c** (a) and **4d** (b); Figure S7. Crystal packing viewed along b-axis for **4d**; Figure S8. View of the asymmetric unit (large atoms and bonds radii) showing its interaction with adjacent molecules for **4b** (a) and **4e** (b); Figure S9. Crystal packing viewed along b-axis for compounds **4b** (a) and **4e** (b); Figure S10. Powder XRD of compounds **4b** and **4c**; Table S1. Crystal data and details of structure refinement for **4b**, **4c**, **4d** and **4e**; Table S2. Bond distances and angles for **4b**; Table S3. Bond distances and angles for **4c**; Table S4. Bond distances and angles for **4d**; Table S5. Bond distances and angles for **4e**.
