# Supplementary material for: New Library of Iodo-Quinoline Derivatives Obtained by an Alternative Synthetic Pathway and Their Antimicrobial Activity
Source: Molecules. 2024 Feb 7;29(4):772. doi: 10.3390/molecules29040772 (PMC10891861; doi:10.3390/molecules29040772)
Supplement: Supplementary file 1 [file molecules-29-00772-s001.zip › molecules-2844158-supplementary.pdf]

Supporting Information

New library of Iodo-quinoline derivatives obtained by an alternative synthetic pathway and their antimicrobial activity.

Cristina M. Al-Matarneh[1,2*], Alina Nicolescu[3], Ioana C. Marinas[2], Madalina D. Gaboreanu[2], Sergiu Shova[4], Andrei Dascalu[1], Mihaela Silion[5], Mariana Pinteala[1]

[1]Center of Advanced Research in Bionanoconjugates and Biopolymers, "Petru Poni" Institute of Macromolecular Chemistry of Romanian Academy, 41A Grigore Ghica Voda Alley, Iasi 700487, Romania;

[2]Research Institute of the University of Bucharest-ICUB, 91-95 Spl. Independentei, 050095 Bucharest, Romania;

[3]NMR Laboratory "Petru Poni" Institute of Macromolecular Chemistry of Romanian Academy, 41A Grigore Ghica Voda Alley, Iasi 700487, Romania;

[4]Department of Inorganic Polymers "Petru Poni" Institute of Macromolecular Chemistry of Romanian Academy, 41A Grigore Ghica Voda Alley, Iasi 700487, Romania;

[5]Physics of Polymers and Polymeric Materials Department, "Petru Poni" Institute of Macromolecular Chemistry, 41A Grigore Ghica Voda Alley, 700487, Iasi, Romania;

almatarneh.cristina@icmpp.ro;

Table of Contents

Figure S1.A. The ^{1}H-NMR spectrum corresponding to compound **4c**, recorded in DMSO-d$_6$, at 400.1 MHz. 5

Figure S1.B. The ^{13}C-NMR spectrum corresponding to compound **4c**, recorded in DMSO-d$_6$, at 100.6 MHz. 5

Figure S1.C. Detailed region of the H,C-HSQC spectrum corresponding to compound **4c**, showing the correlation signals for protonated carbons. 6

Figure S1.D. The H,C-HMBC spectrum corresponding to compound **4c**, showing 2 or 3 bonds correlation signals between protons and carbons, used mainly to assign quaternary carbons...... 7

Figure S1.E. MALDI-MS spectra of compound **4c**. 8

Figure S1.F. FT-IR spectrum of compound **4c**. 8

Figure S2.A. The ^{1}H-NMR spectrum corresponding to compound **4t**, recorded in DMSO-d$_6$, at 400.1 MHz. 9

Figure S2.B. The ^{13}C-NMR spectrum corresponding to compound **4t**, recorded in DMSO-d$_6$, at 100.6 MHz. 9

Figure S2.C. MALDI-MS spectrum of the compound **4t**. 10

Figure S2.D. FT-IR spectrum of compound **4t.** 10

Figure S3.A. The ^{1}H-NMR spectrum corresponding to compound **8c**, recorded in DMSO-d$_6$, at 400.1 MHz. 11

Figure S3.B. MALDI-MS spectra of compound **8c**. 11

Figure S3.C. FT-IR spectrum of compound **8c.** 12

Figure S4.A. The ^{1}H-NMR spectrum corresponding to compound **5n**, recorded in DMSO-d$_6$, at 400.1 MHz. 13

Figure S4.B. The ^{13}C-NMR spectrum corresponding to compound **5n**, recorded in DMSO-d$_6$, at 100.6 MHz. 14

Figure S4.C. The H,C-HMBC spectrum corresponding to compound **5n**, recorded in DMSO-d$_6$, showing correlation signals between vinylic protons and either quinoline's CH-3 or benzaldehyde's C-14 carbon atoms. 15

Figure S4.D. Maldi-MS spectrum corresponding to compound **5n**. 15

Figure S4.E. FT-IR spectrum of compound **5n**. 16

Figure S5.A. The ^{1}H-NMR spectrum corresponding to compound **7**, recorded in DMSO-d$_6$, at 600.1 MHz. 17

Figure S5.B. The ^{13}C-NMR spectrum corresponding to compound **7,** recorded in DMSO-d_6, at 150.9 MHz. 18

Figure S5.C. Maldi-MS spectrum corresponding to compound **7**..................18

Figure S5.D. FT-IR spectrum of compound **7.**..................18

Figure S6. View of the asymmetric unit (large atoms and bonds radii) showing its interaction with adjacent molecules for **4c** (a) and **4d** (b)..................19

Figure S7. Crystal packing viewed along *b* axis for **4d**...................20

Figure S8. View of the asymmetric unit (large atoms and bonds radii) showing its interaction with adjacent molecules for **4b** (a) and **4e** (b). 20

Figure S9. Crystal packing viewed along *b* axis for compounds **4b** (a) and **4e** (b). 21

Figure S10. Powder XRD of compounds **4b** and **4c**...................22

Table S1. Crystal data and details of structure refinement for **4b, 4c, 4d** and **4e**...................23

Table S2. Bond distances and angles for **4b** 23

Table S3. Bond distances and angles for **4c**. 24

Table S4. Bond distances and angles for **4d**. 25

Table S5. Bond distances and angles for **4e**...................26

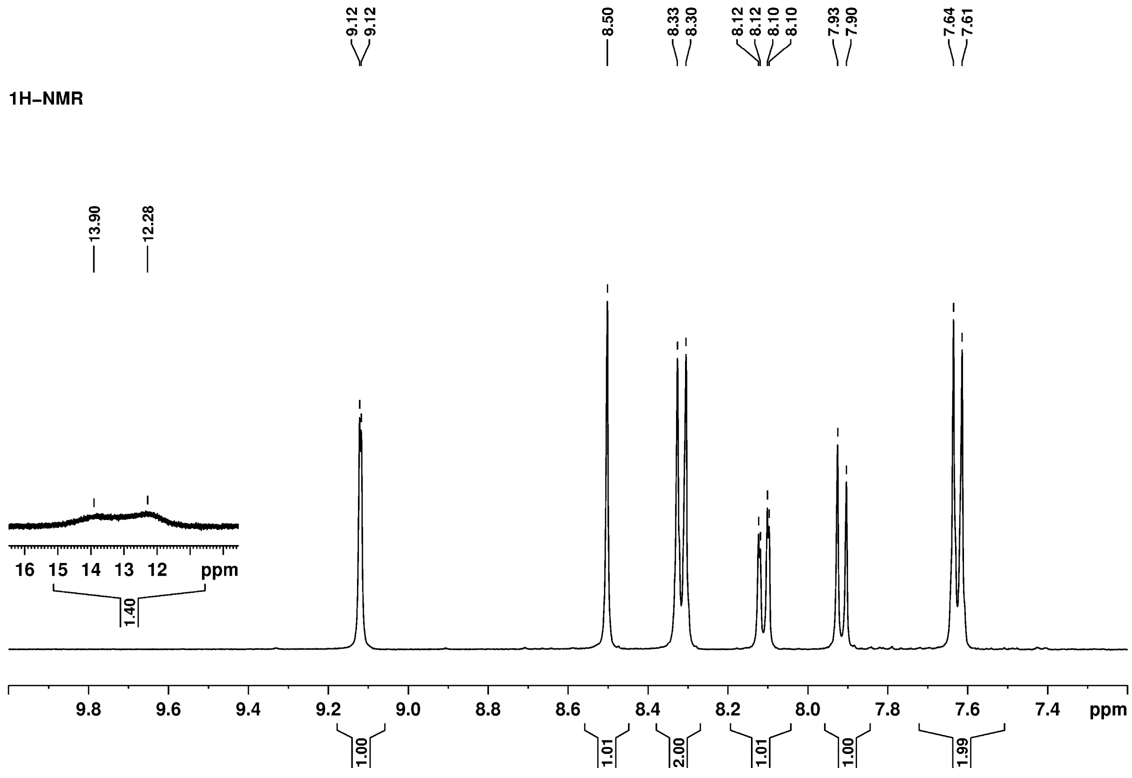

Figure S1.A. The ^{1}H-NMR spectrum corresponding to compound **4c**, recorded in DMSO-d$_6$, at 400.1 MHz.

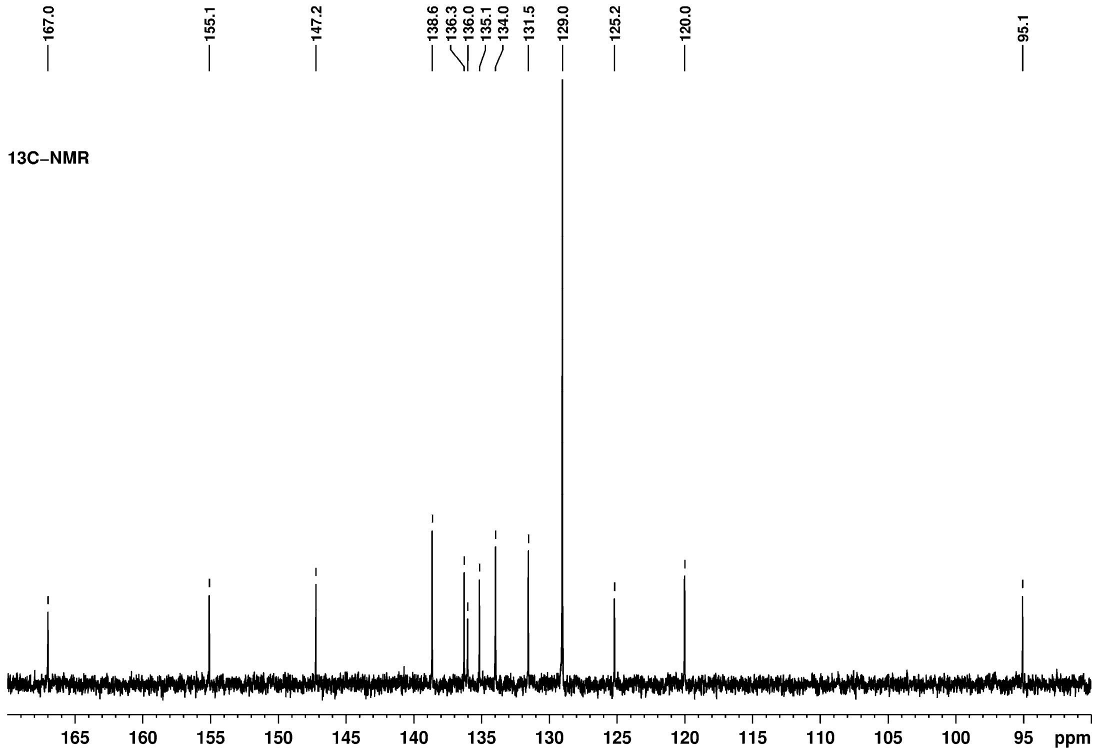

Figure S1.B. The ^{13}C-NMR spectrum corresponding to compound **4c**, recorded in DMSO-d_6, at 100.6 MHz.

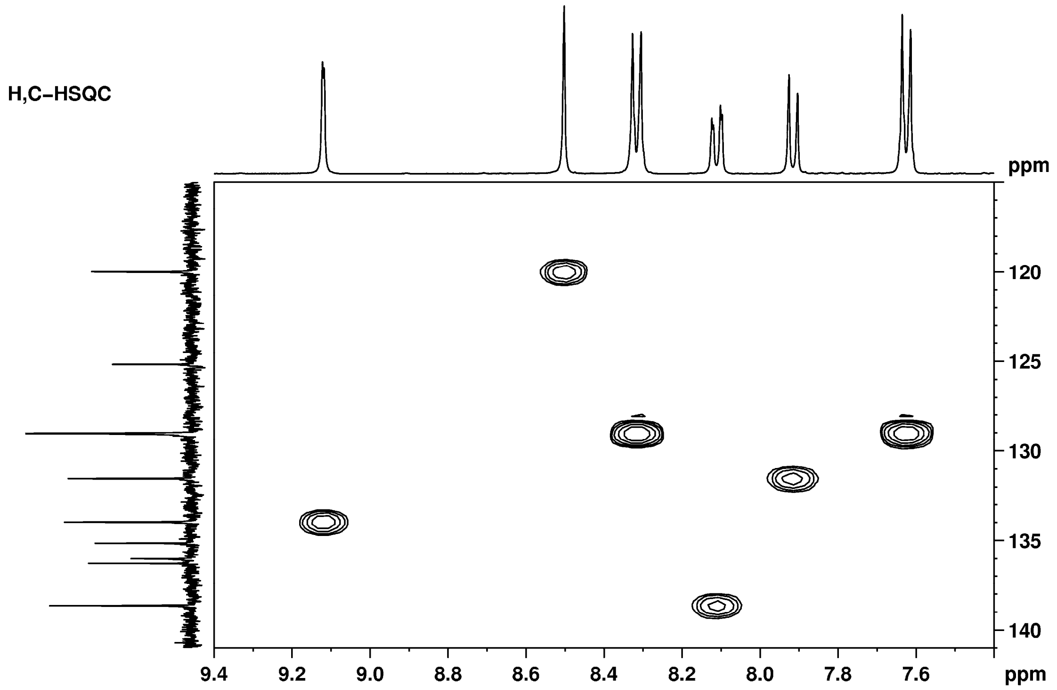

Figure S1.C. Detailed region of the H,C-HSQC spectrum corresponding to compound **4c**, showing the correlation signals for protonated carbons.

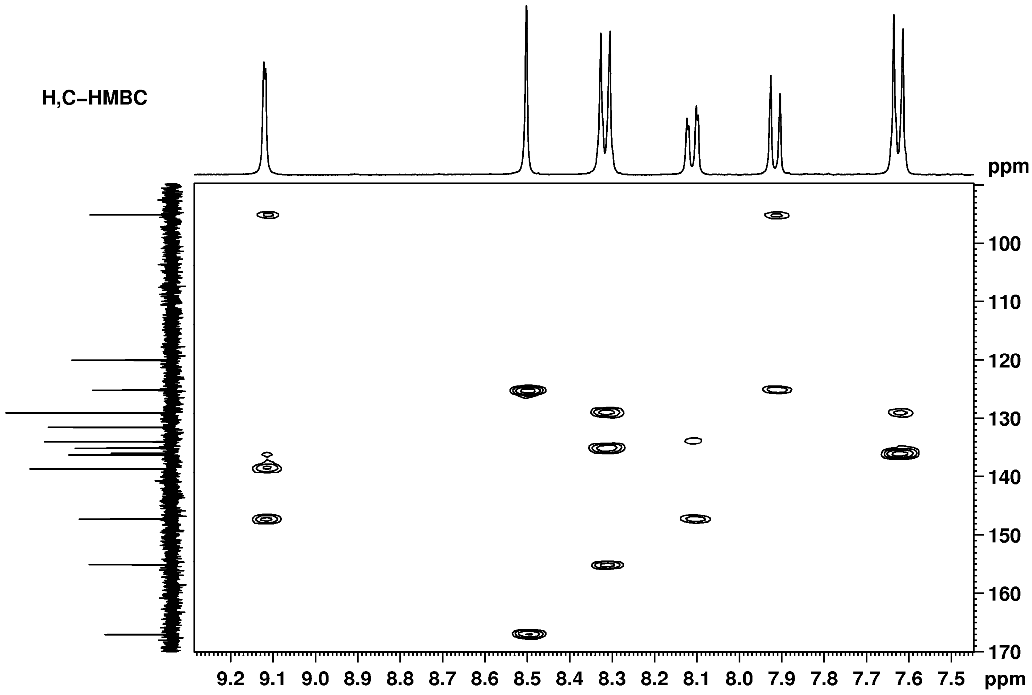

Figure S1.D. The H,C-HMBC spectrum corresponding to compound **4c**, showing 2 or 3 bonds correlation signals between protons and carbons, used mainly to assign quaternary carbons.

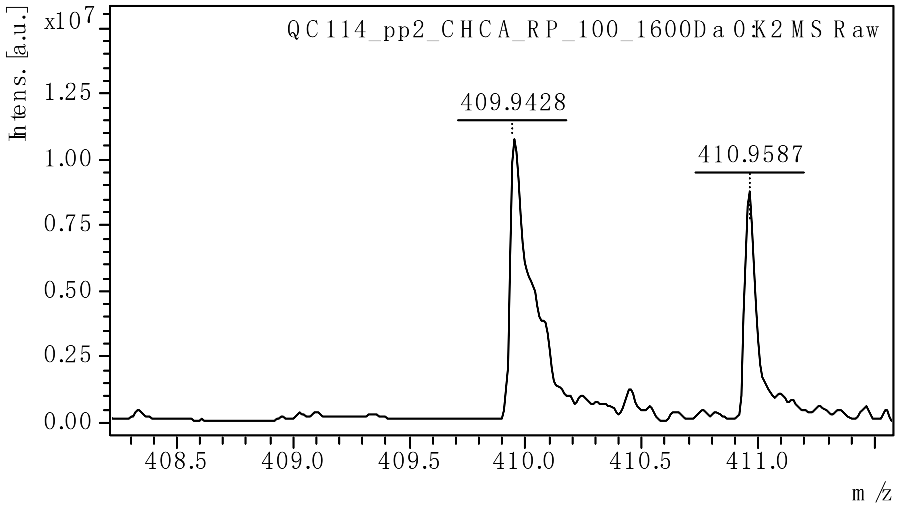

Figure S1. E. MALDI-MS spectra of compound **4c**.

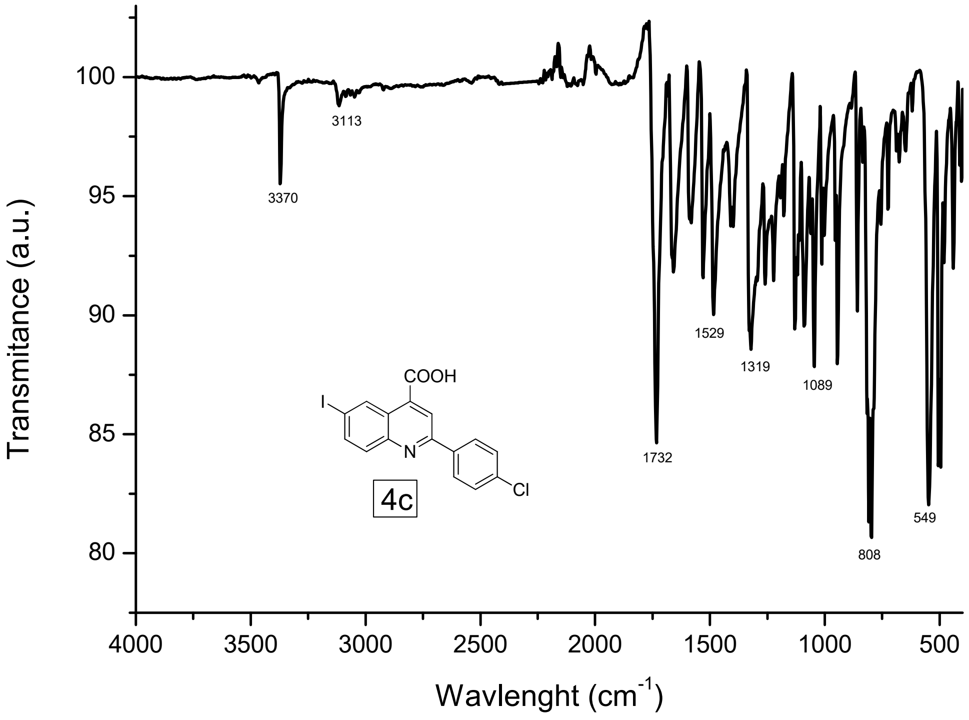

Figure S1.F. FT-IR spectrum of compound **4c**.

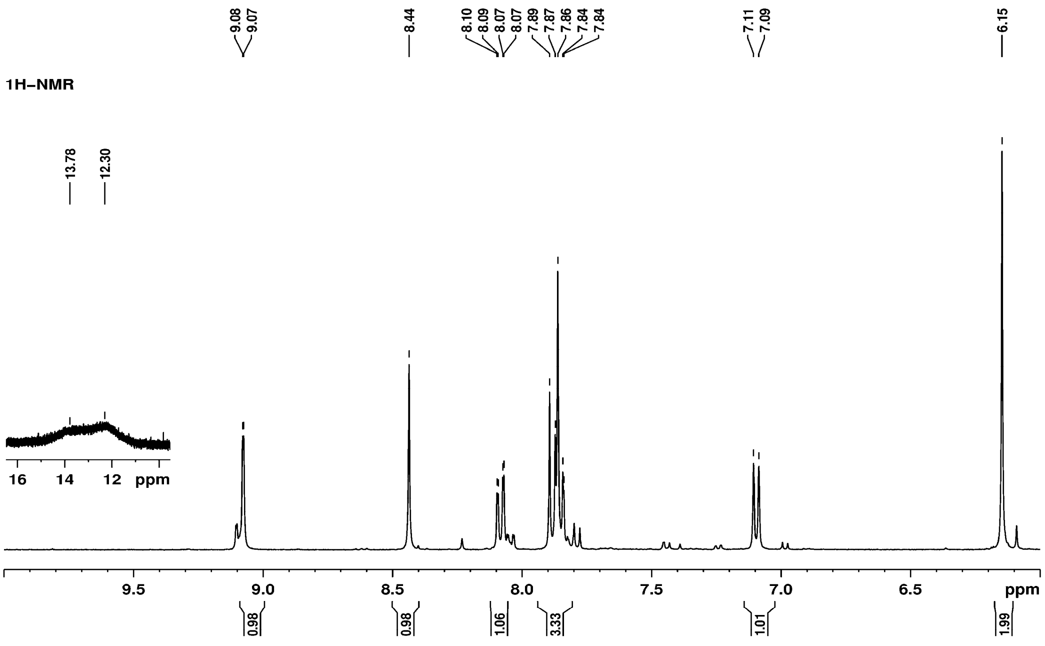

Figure S2.A. The ^{1}H-NMR spectrum corresponding to compound **4t**, recorded in DMSO-d_6, at 400.1 MHz.

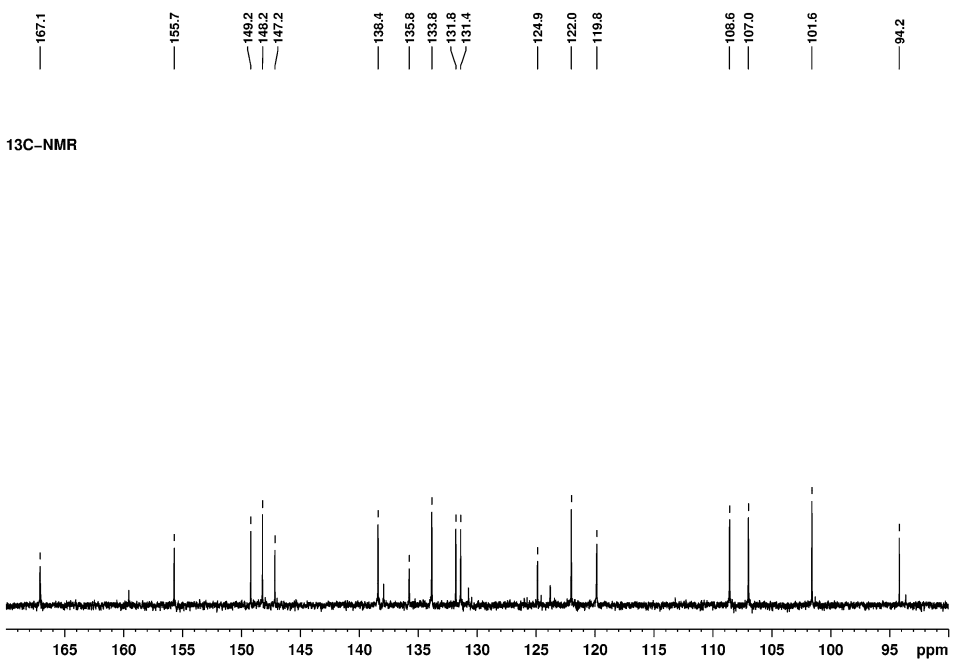

Figure S2.B. The ^{13}C-NMR spectrum corresponding to compound **4t**, recorded in DMSO-d_6, at 100.6 MHz.

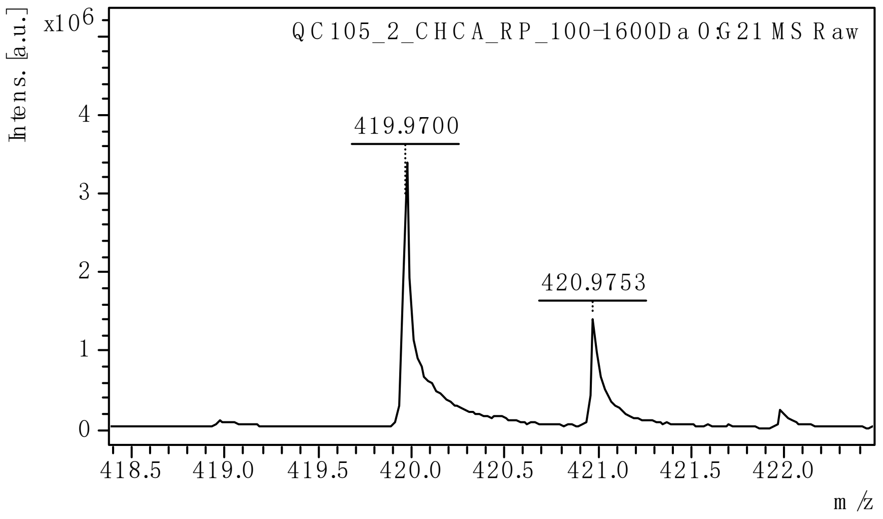

Figure S2.C. MALDI-MS spectrum of the compound **4t**.

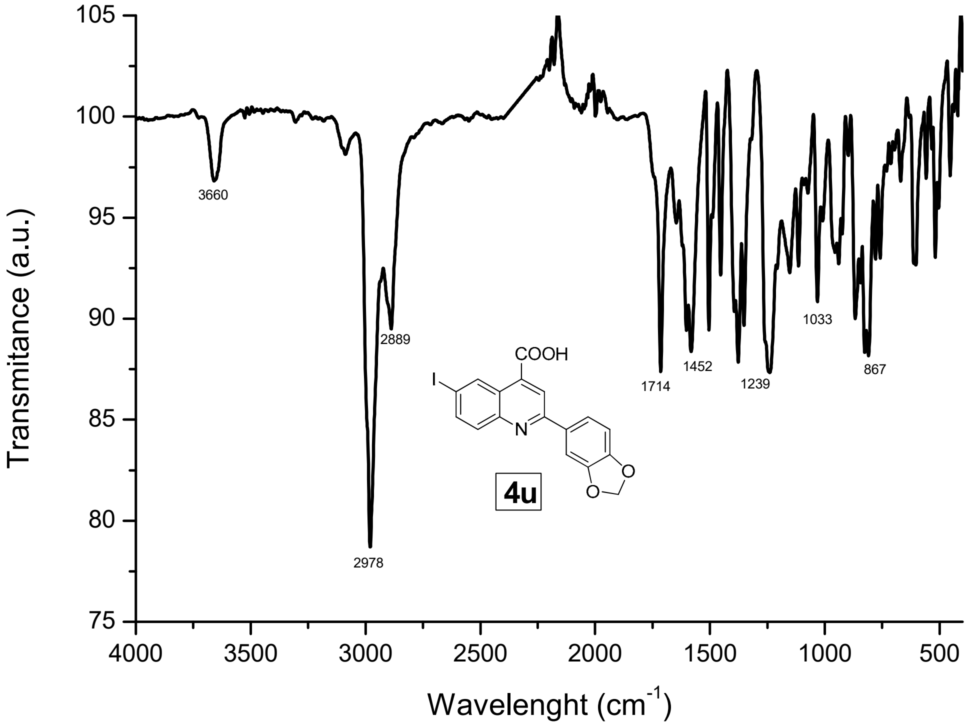

Figure S2.D. FT-IR spectrum of compound **4t.**

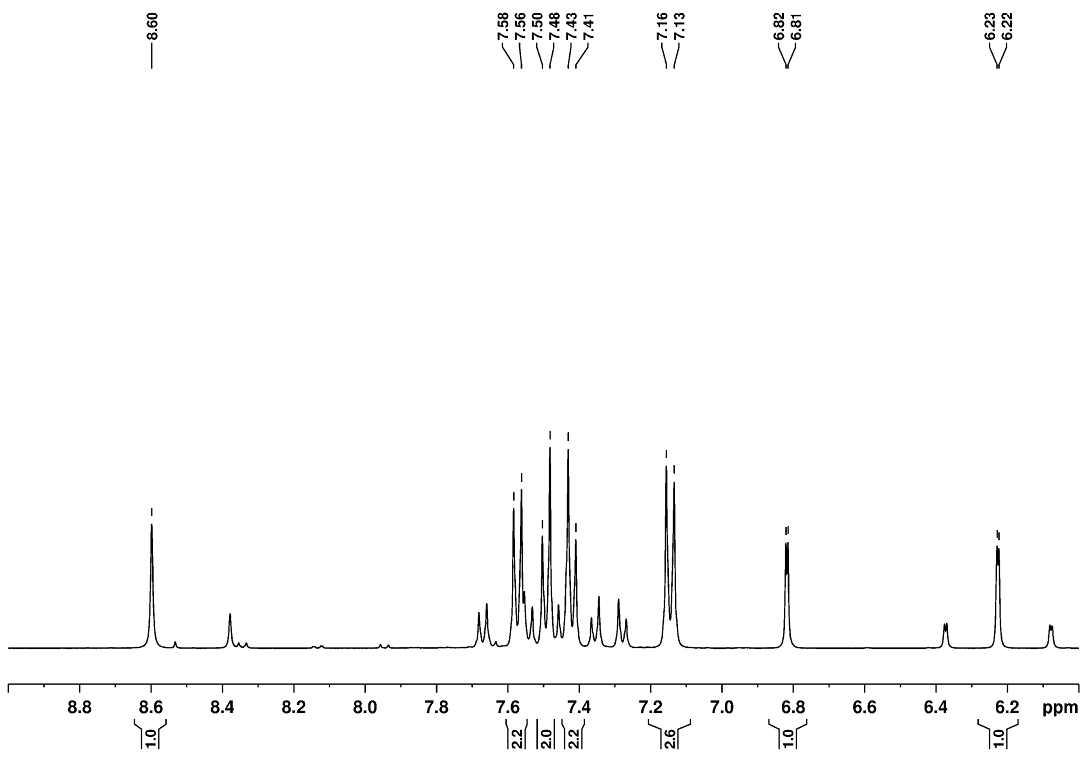

Figure S3.A. The ^{1}H-NMR spectrum corresponding to compound **8c**, recorded in DMSO-d_6, at 400.1 MHz.

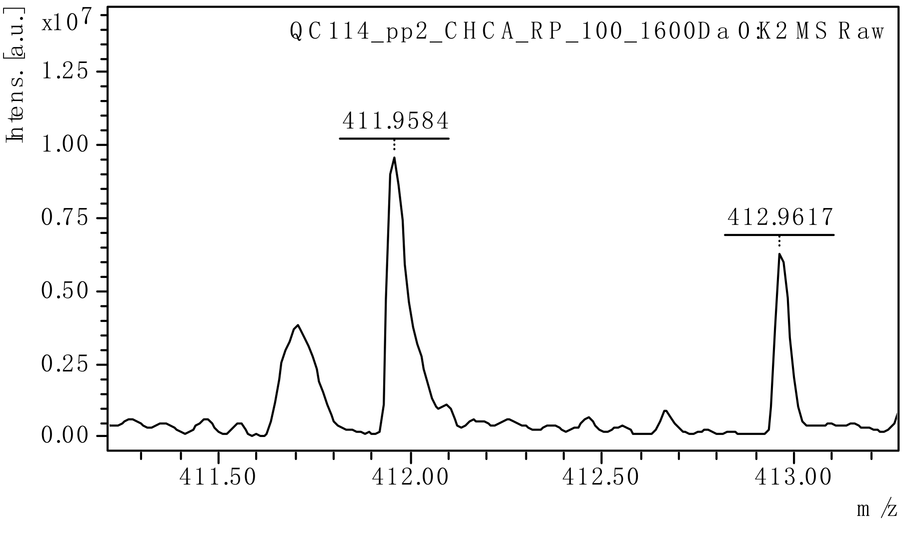

Figure S3.B. MALDI-MS spectra of compound **8c**.

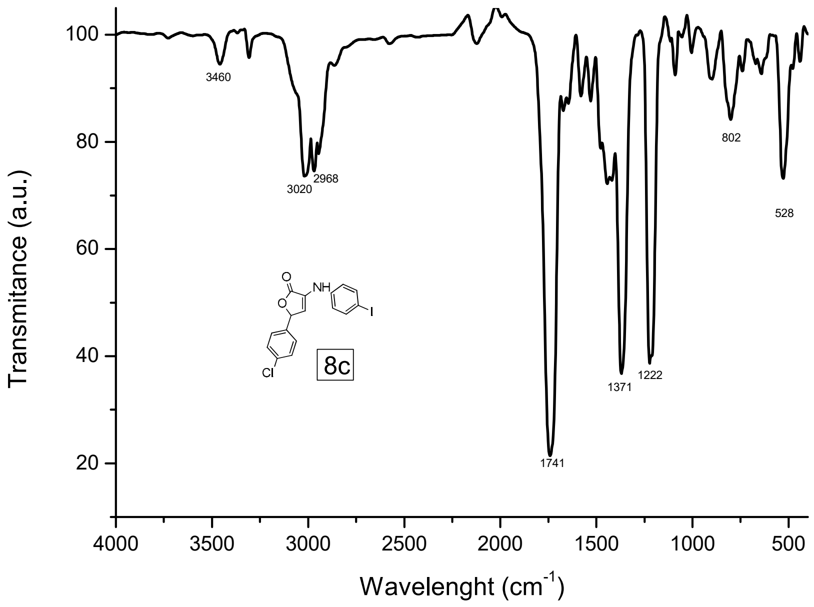

Figure S3.C. FT-IR spectrum of compound **8c.**

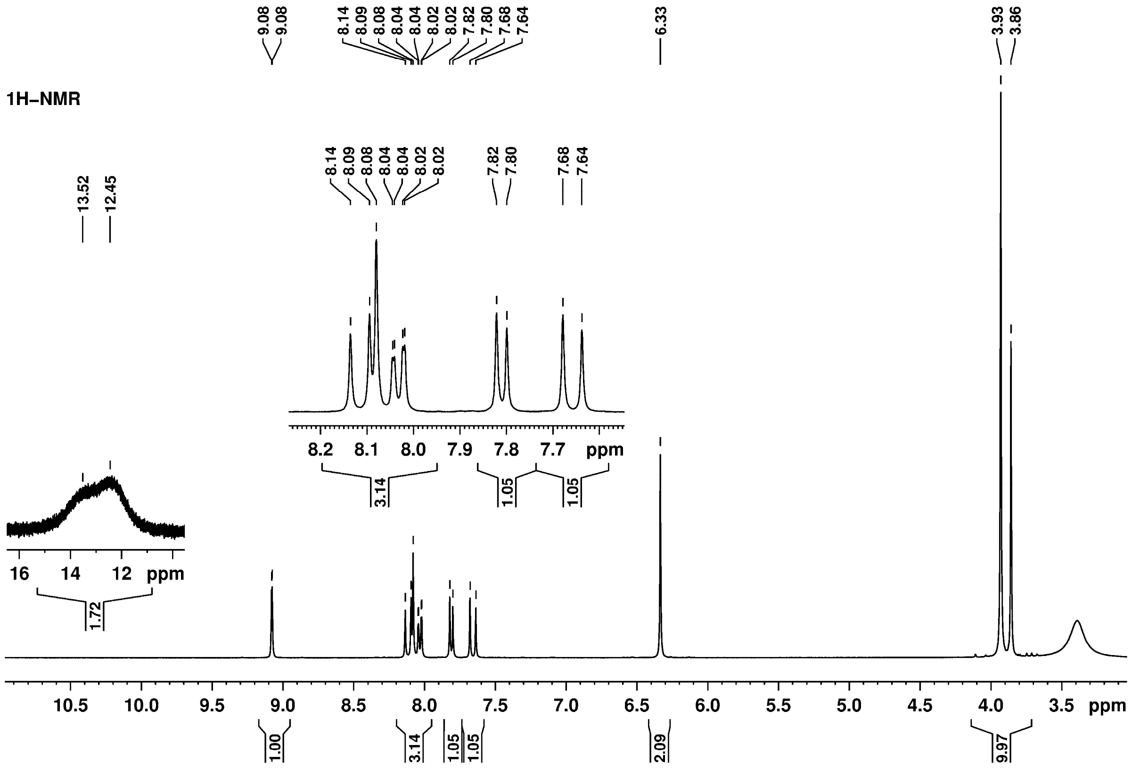

Figure S4.A. The ^{1}H-NMR spectrum corresponding to compound **5n**, recorded in DMSO-d_6, at 400.1 MHz.

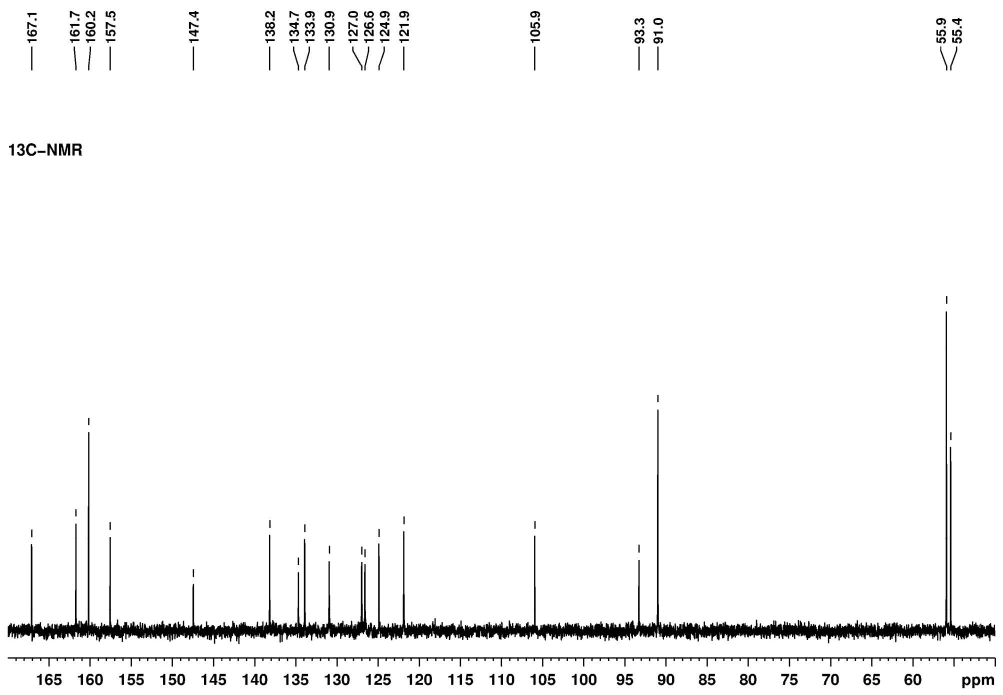

Figure S4.B. The ^{13}C-NMR spectrum corresponding to compound **5n**, recorded in DMSO-d6, at 100.6 MHz.

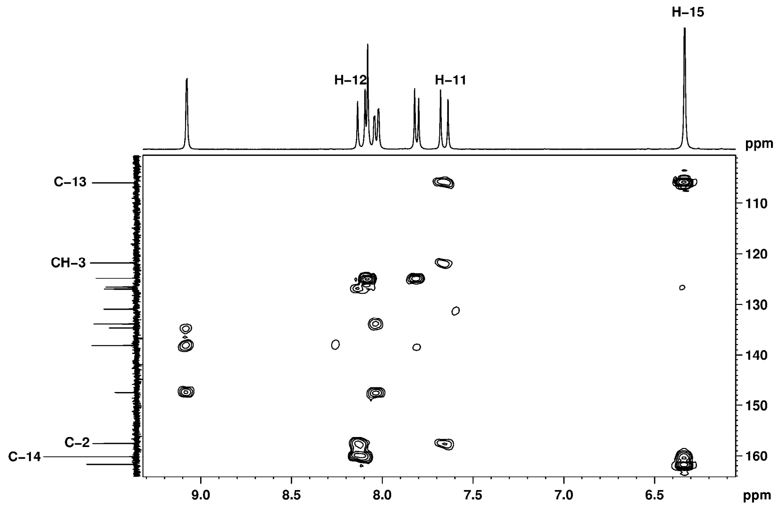

Figure S4.C. The H,C-HMBC spectrum corresponding to compound **5n**, recorded in DMSO-d6, showing correlation signals between vinylic protons and either quinoline's CH-3 or benzaldehyde's C-14 carbon atoms.

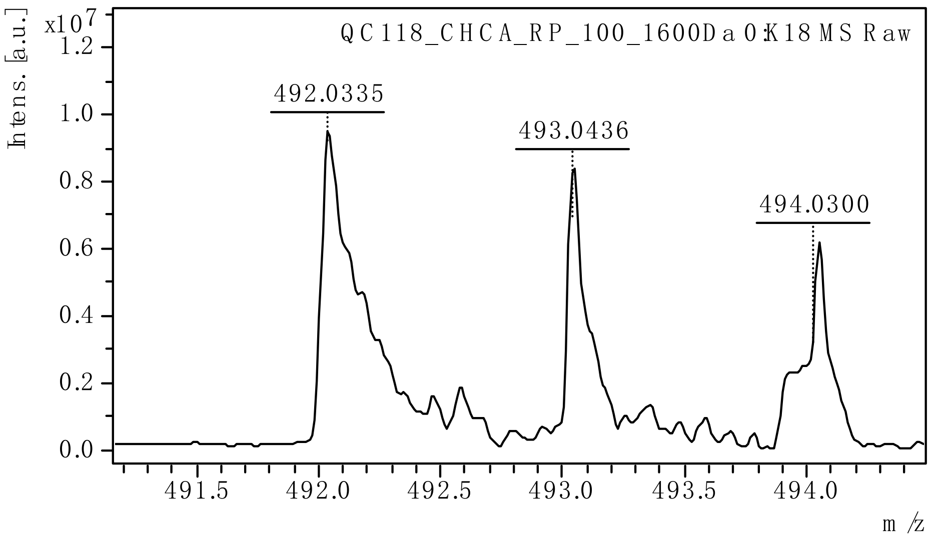

Figure S4.D. Maldi-MS spectrum corresponding to compound **5n**.

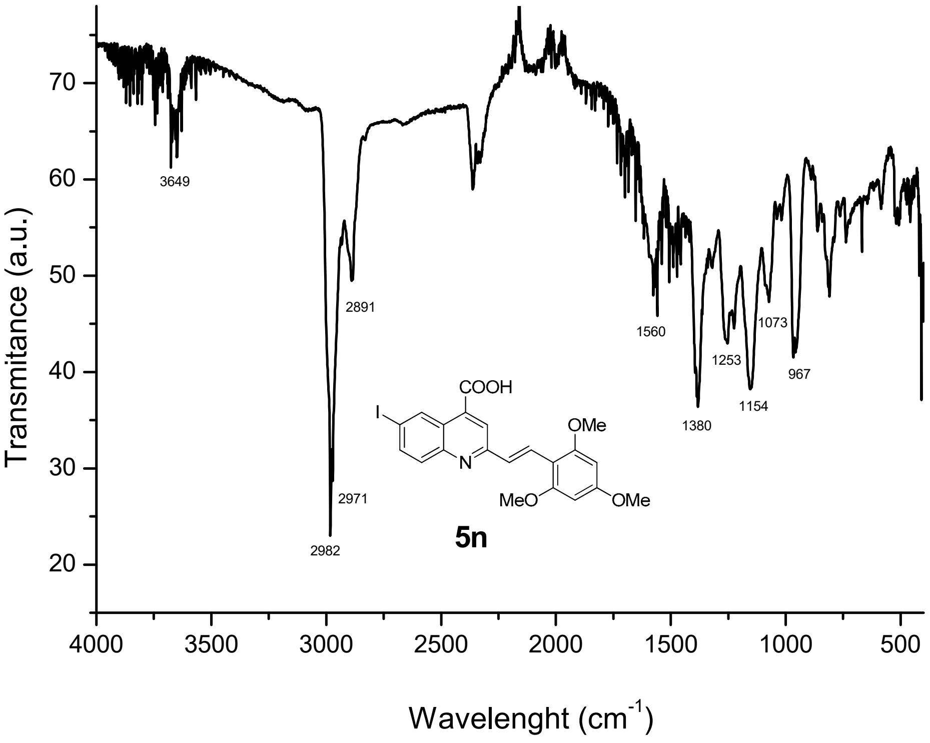

Figure S4.E. FT-IR spectrum of compound **5n**.

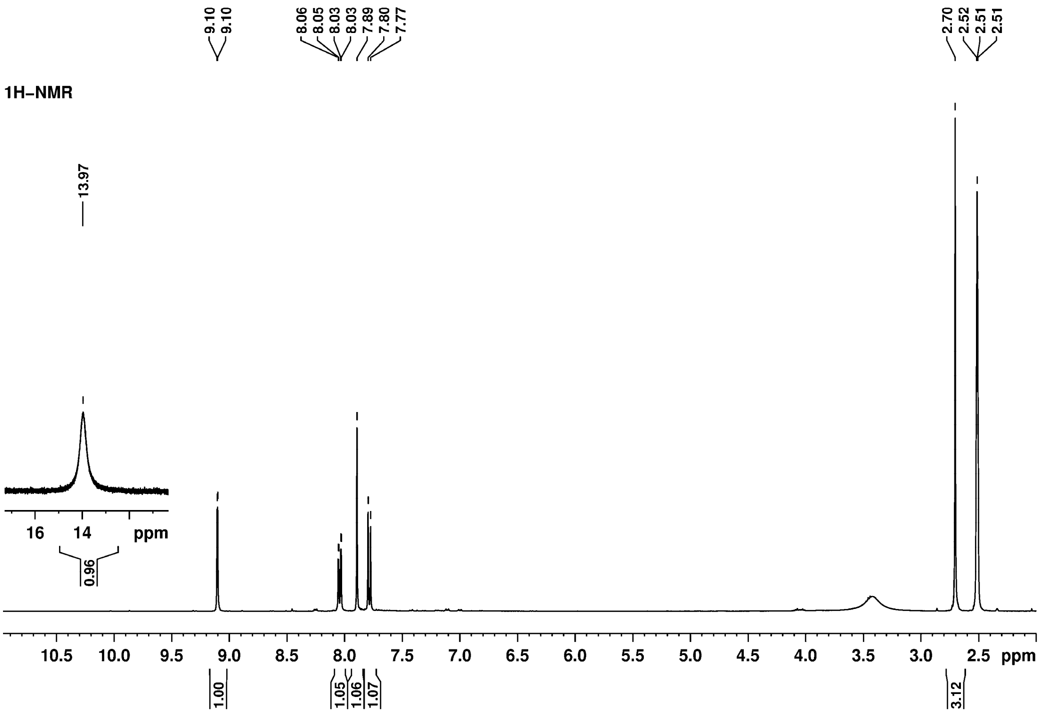

Figure S5.A. The ^{1}H-NMR spectrum corresponding to compound **7**, recorded in DMSO-d_6, at 600.1 MHz.

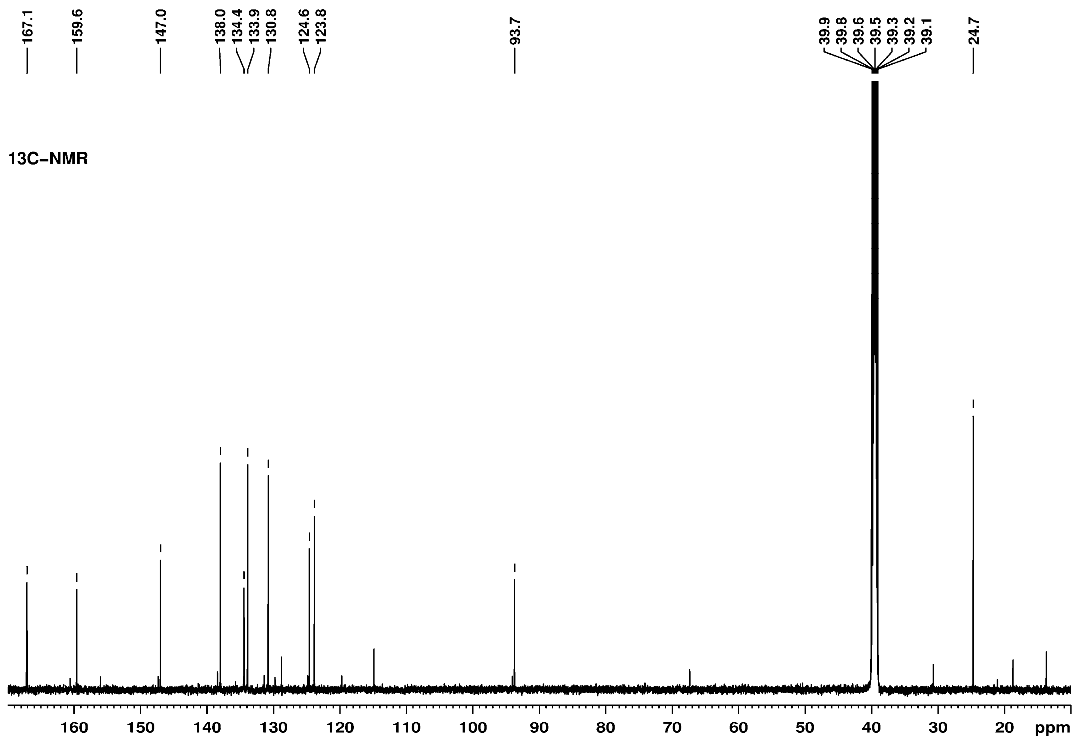

Figure S5.B. The ^{13}C-NMR spectrum corresponding to compound **7,** recorded in DMSO-d_6, at 150.9 MHz.

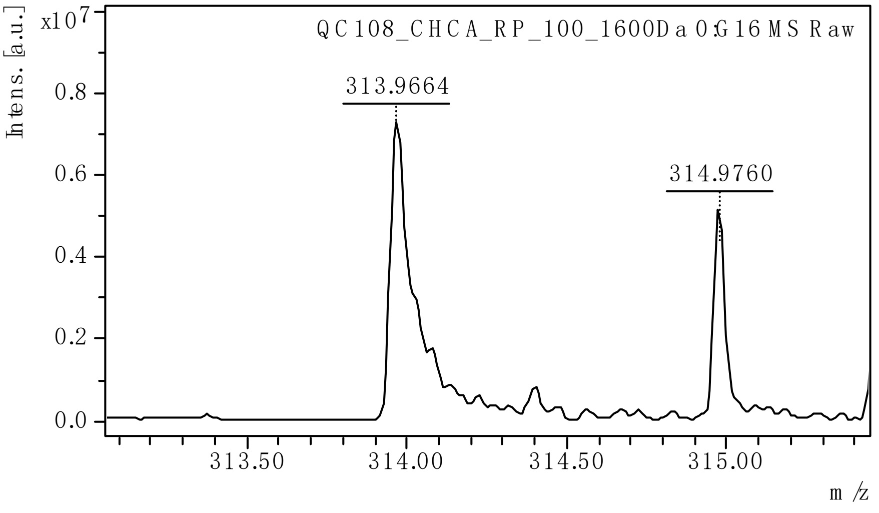

Figure S5.C. Maldi-MS spectrum corresponding to compound **7**.

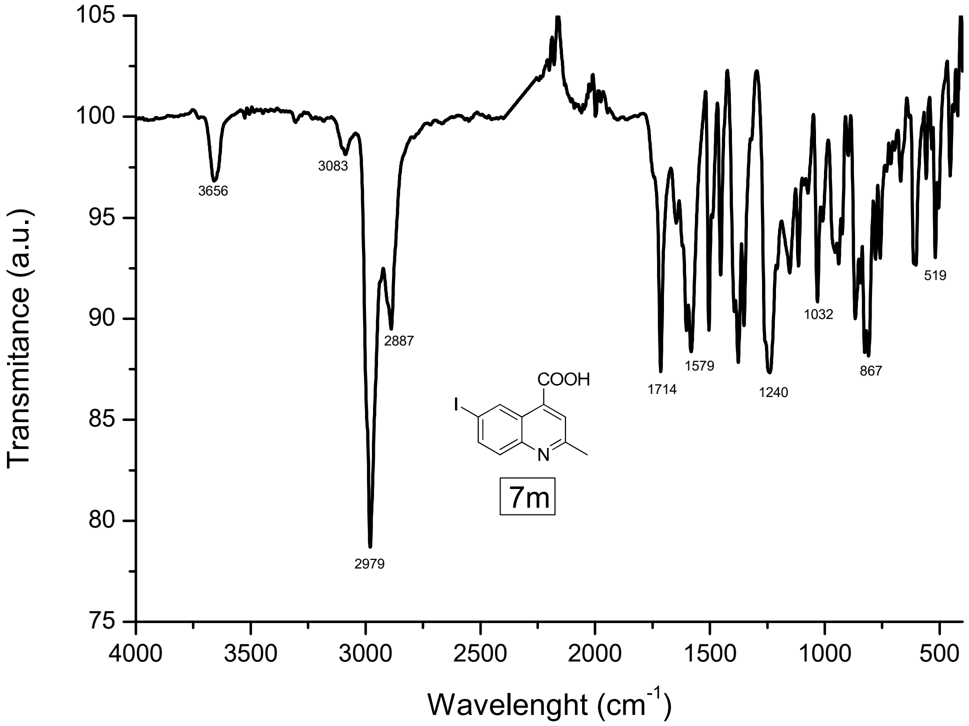

Figure S5.D. FT-IR spectrum of compound **7.**

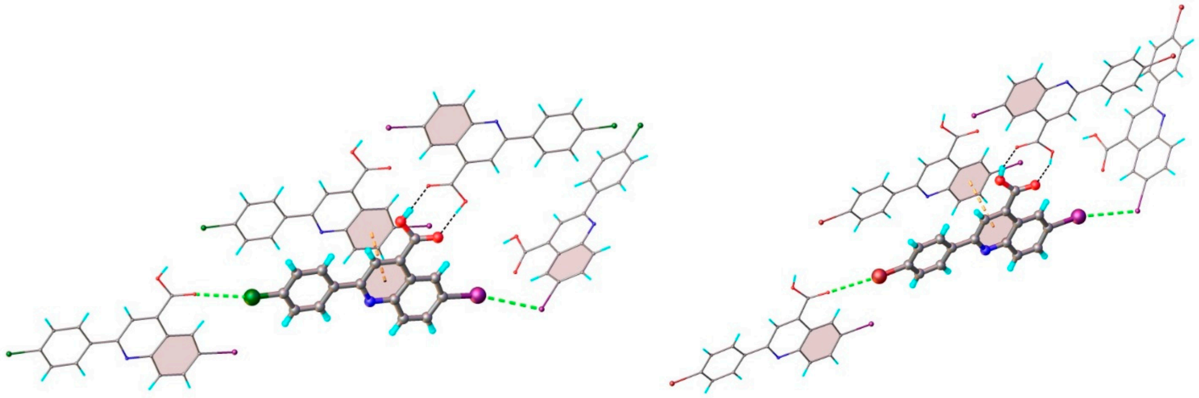

a) b)

Figure S6. View of the asymmetric unit (large atoms and bonds radii) showing its interaction with adjacent molecules for **4c** (a) and **4d** (b). Hydrogen and halogen bonds are shown as dotted lines with black and green color, respectively.

H-bonds parameters:

For **4c**: O2-H---O1 [O2-H 0.84 Å, H···O1($-x$, $-y$, $1-z$) 1.84 Å, O2···O1 2.683(3) Å, ∠O2HO1 177.2°.

For **4d**: O2-H---O1 [O2-H 0.86 Å, H···O1($-x$, $-y$, $1-z$) 1.85 Å, O2···O1 2.674(6) Å, ∠O2HO1 160.6°.

For **4c**: [C14-Cl1···O1] C14-Cl1 1.744(1) Å, Cl1···O1(1 – x, $y-1$, z) 3.211(1) Å, ∠C14Cl1O1 169.3(1)°.

Halogen bond parameters:

For **4d**: [C14-Br1···O1] C14-Br1 1.891(7) Å, Br1···O1(1 – x, $y-1$, z) 3.314(5) Å, ∠C14Br1O1 162.2(4)°.

I···I contacts: 3.8052(1) (for **4c**)Å; 3.9115(8) Å (for **4d**);

Centroid-to-centroid distances (orange dotted lines): 3.5512 Å (for **4c**); 3.5878(1) Å (for **4d**);

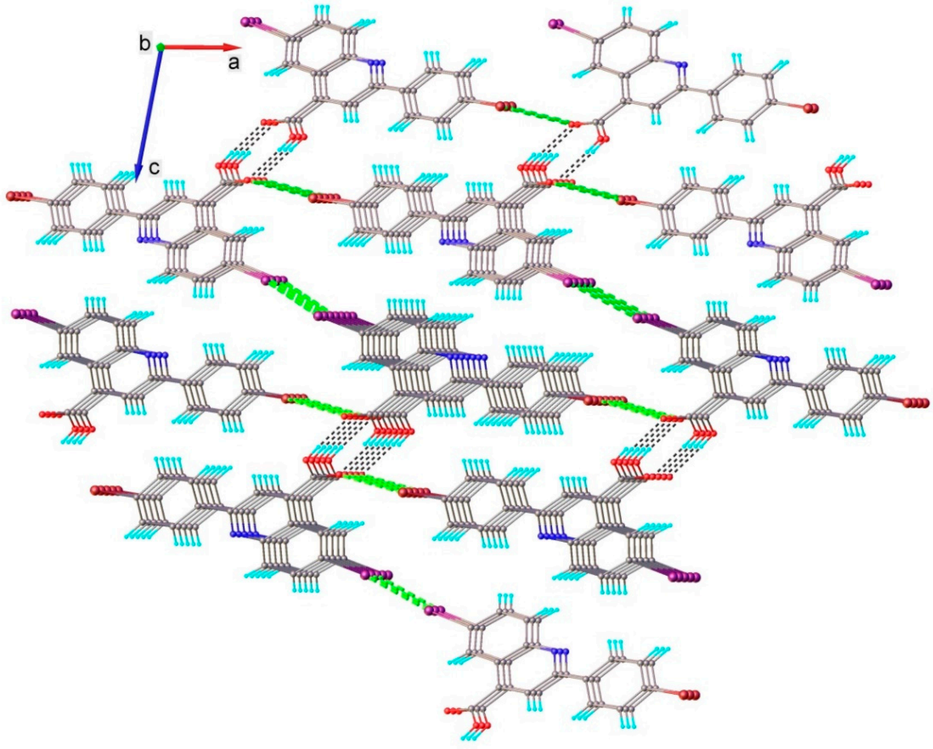

Figure S7. Crystal packing viewed along *b* axis for **4d**.
